# Transition from stromatolite to thrombolite fabric: potential role for reticulopodial protists in lake microbialites of a Proterozoic ecosystem analog

**DOI:** 10.3389/fmicb.2023.1210781

**Published:** 2023-10-30

**Authors:** Joan M. Bernhard, Luke A. Fisher, Quinne Murphy, Leena Sen, Heidi D. Yeh, Artemis Louyakis, Fatma Gomaa, Megan Reilly, Paola G. Batta-Lona, Ann Bucklin, Veronique Le Roux, Pieter T. Visscher

**Affiliations:** ^1^Department of Geology and Geophysics, Woods Hole Oceanographic Institution, Woods Hole, MA, United States; ^2^Department of Marine Sciences, University of Connecticut, Groton, CT, United States; ^3^Department of Earth and Atmospheric Sciences, Cornell University, Ithaca, NY, United States; ^4^Department of Marine and Coastal Sciences, Rutgers University, New Brunswick, NJ, United States; ^5^Department of Molecular and Cell Biology, University of Connecticut, Storrs, CT, United States; ^6^Department of Organismic and Evolutionary Biology, Harvard University, Cambridge, MA, United States; ^7^Department of Marine Sciences, Northeastern University, Boston, MA, United States

**Keywords:** microbialite, stromatolite, Fayetteville Green Lake, Rhizaria, *Chlamydomyxa labyrinthuloides*, *Haplomyxa saranae*, foraminifera, paleomicrobiology

## Abstract

Prior observations suggest that foraminiferan protists use their reticulopodia (anastomosing pseudopodia) to alter sediment fabric by disrupting laminations of subtidal marine stromatolites, erasing the layered structures in an experimental setting. Because microbialites and foraminifera are found in non-marine settings, we hypothesized that foraminifera living in lakes could also disrupt layered microbialite fabric. With this aim and using a variety of multidisciplinary approaches, we conducted field surveys and an experiment on microbialites from Green Lake (GL; Fayetteville, New York State, United States), which has been studied as a Proterozoic ecosystem analog. The lake is meromictic and alkaline, receiving calcium sulfate-rich water in the monimolimnion; it supports a well-developed carbonate platform that provides access to living and relict microbialites. The living microbialites grow from early spring to autumn, forming a laminated mat at their surface (top ~5 mm), but a clotted or massive structure exists at depth (> ~ 1 cm). We observed a morphotype of “naked” foraminiferan-like protist in samples from GL microbialites and sediments; thus, considered the possibility of freshwater foraminiferan impact on microbialite fabric. Results of an experiment that seeded the cultured freshwater foraminifer *Haplomyxa saranae* onto the GL microbialite surface indicates via micro-CT scanning and anisotropy analysis that the introduced foraminifer impacted uppermost microbialite layering (*n* = 3 cores); those cores with an added inhibitor lacked changes in anisotropy for two of those three cores. Thus, it remains plausible that the much smaller, relatively common, native free-form reticulate protist, which we identified as *Chlamydomyxa labyrinthuloides*, can disrupt microbialite fabrics on sub-millimeter scales. Our observations do not exclude contributions of other possible causal factors.

## 1. Introduction

Lithifying microbial mats or microbialites (Burne and Moore, 1987), the oldest dated at >3.4 billion years, are the most visible manifestations of pervasive microbial life on early Earth (Allwood et al., 2006; Nutman et al., 2016). Changes in microbialite abundance and morphology over time document complex interplays between biological, geological, and chemical processes (Riding, 2000). Microbialites can be classified as one of three main types based on their mesostructure (visible with naked eyes; Dupraz et al., 2009): stromatolite (laminated), thrombolite (clotted), and leiolite (structureless). Details of fossil stromatolite formation and preservation are controversial: A number of factors are involved in microbialite framework construction, including carbonate saturation state, sediment dynamics, and metazoan, algal, and cyanobacterial abundance and activities (Shapiro et al., 1995; Visscher et al., 1998; Riding, 2000; Riding and Liang, 2005; Dupraz et al., 2009; Planavsky and Ginsburg, 2009; Tarhan et al., 2013).

A popular hypothesis to explain the late Neoproterozoic decline in stromatolites is the radiation of eukaryotic predators (Walter and Heys, 1985) that consumed or disrupted the microbialites in some manner. The most commonly-proposed predators are unspecified Metazoa (multicelluar eukaryotes; e.g., Planavsky and Ginsburg, 2009), although fossil evidence to support this perspective is nonexistent. Protists (single-celled eukaryotes) that lack agglutinated or mineralized shells are also possible stromatolite predators. However, they are not expected to leave obvious fossils and are largely ignored in this context.

Prior observations suggest reticulopods of the Rhizarian protistan phylum Foraminifera can alter microbialite fabric by disrupting laminations of subtidal marine stromatolites, changing the layered (stromatolitic) structures into leiolites in an experimental setting (Bernhard et al., 2013). A Proterozoic origin of foraminifera is likely given their earliest fossils are Cambrian (Culver, 1991) or even earlier (Bosak et al., 2012), and molecular-clock analyses support a Proterozoic origin of “soft-shelled” foraminifera (Pawlowski et al., 2003). Because of this Precambrian origin and foraminiferal occurrence in non-marine settings (Holzmann et al., 2003, 2021; Siemensma et al., 2021), we hypothesized that freshwater foraminifera impacted the fabric of lake stromatolites. Here we present geochemical results and observations on benthic microbial eukaryotes from Green Lake (GL; Fayetteville, NY, United States) microbialites, as well as results from an experiment to assess impact of freshwater foraminifera on layered GL microbialite fabric. While the prokaryotic assemblages of the GL microbialites have been described, especially the cyanobacteria (Wilhelm and Hewson, 2012), to our knowledge very little has been published on the microscopic eukaryotes of the platform. Studying if and how protist communities affect the development of modern microbialites is critical to interpretations of the fossil record, especially in the context of Proterozoic ecosystems.

**Figure 1:**
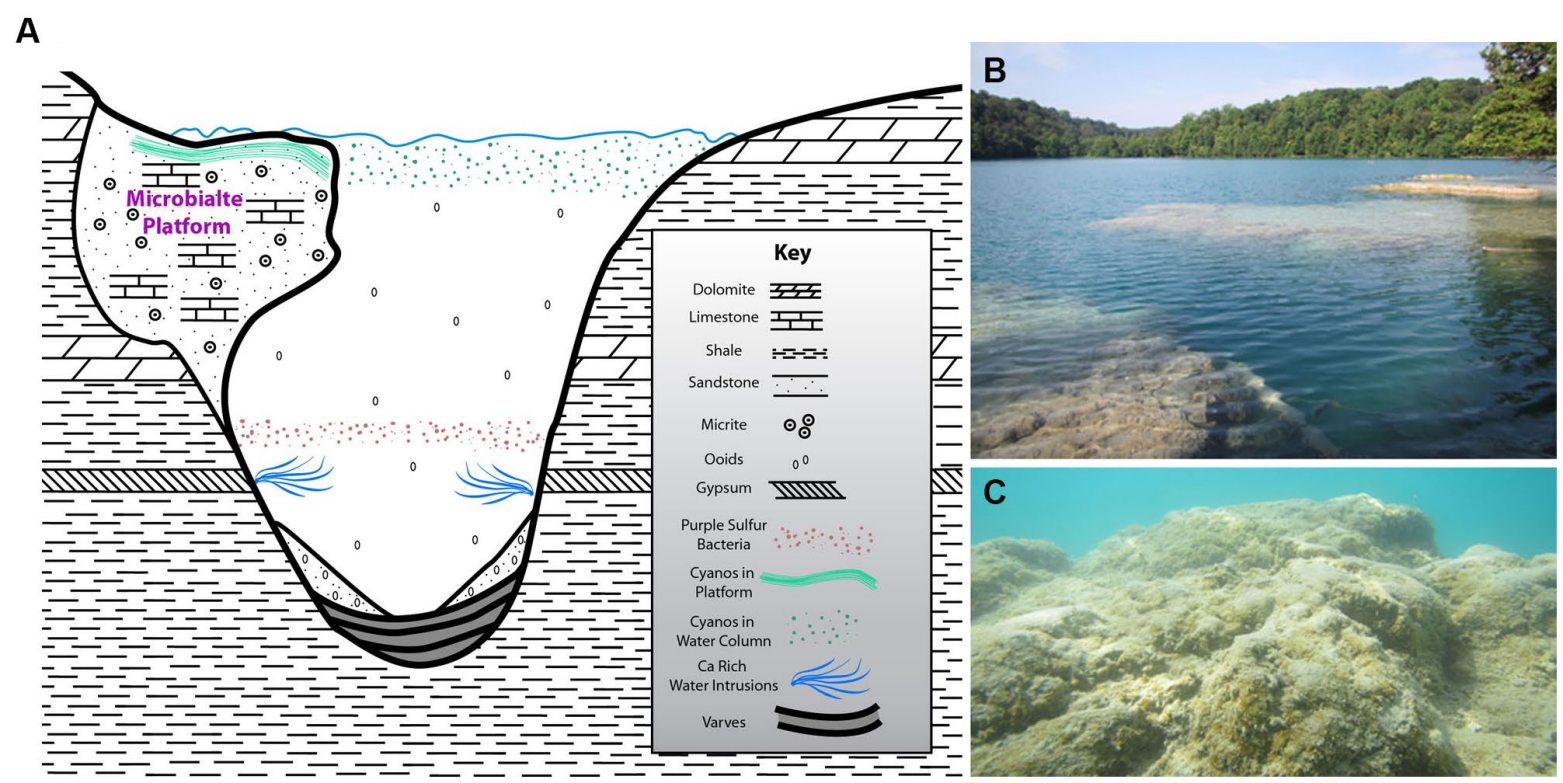
**(A)** Schematic cross section of the meromictic alkaline Green Lake (Fayetteville, NY, United States) including Dead Man’s Point (DMP) microbialite platform (not to scale) and local bedrock formation information, highly modified from Thompson et al. (1990). **(B)** Above-water overview of DMP microbialite platform. **(C)** Underwater view of portion of DMP platform surface, showing some “fluffy” EPS and macrophytes in spots.

Green Lake is an alkaline meromictic lake that serves as an analog to the Proterozoic oceanic ecosystem (Thompson et al., 1990; Havig et al., 2015; Herndon et al., 2018; Kamennaya et al., 2020), which is generally considered to have oxygenated surface waters and anoxic/ sulfidic (euxinic) deep waters at depth (Canfield et al., 2008; Reinhard et al., 2013) and widespread shallow-water stromatolites (Knoll et al., 2016). The lake presently supports a well-developed shallow-water (~ < 1 m) microbialite carbonate platform or bioherm (Figure 1), located at Dead Man’s Point in the north-eastern quadrant of the lake (43.051814 N, 75.963969 W). A smaller platform occurs along a short section of shoreline in the southwestern sector of the lake; we did not investigate this smaller feature.

Green Lake was the result of a meltwater waterfall cascading off a Wisconsin-aged glacier head that eroded a deep hole in the sedimentary bedrock. Thus, the lake is quite deep for its lateral extent, being ~0.25 km wide by ~1.1 km long, but ~59 m deep. The bedrock hosting the lake is largely shale although gypsum, limestone and dolomite also occur further up section (Figure 1A). Ground waters percolating through the bedrock introduce ions into lake waters (e.g., calcium-sulfate-enriched intrusions; Brunskill, 1969; Brunskill and Ludlam, 1969) causing stratification, where depths exceeding about 20 m are strongly reducing (Havig et al., 2018; Block et al., 2021). The bacterial communities of these stratified water masses have been well described (Block et al., 2021).

## 2. Materials and methods

### 2.1. Lake and platform biogeochemistry

Permits were obtained from the New York State Office of Parks, Recreation and Historic Preservation to sample Green Lake. The Dead Man’s Point (DMP) platform and its overlying waters were sampled eight times from May 2017 to November 2019. Physicochemical characteristics of the water overlying the platform were recorded with a Mettler Toledo FiveGo handheld meter (temperature, pH) and an Accumet AP75 conductivity/temperature meter (conductivity). Measurements of the underwater light regime were made approximately 5 cm above the carbonate platform with a quantum meter (LiCor 250) equipped with an underwater PAR quantum sensor (LiCor 192). Water samples (250 mL) were gravity-filtered through 0.22 μm cellulose nitrate filters. One aliquot was transferred to an acid-cleaned screwcap tube and stored on ice for calcium measurement in the laboratory using ion chromatography (Dionex IC3000). To determine alkalinity, a 40-ml aliquot of the filtrate was titrated against 0.1 N HCl at the site. The titration data were entered into USGS Alkalinity Titrator software.^1^

Geochemical depth profiles of oxygen, hydrogen sulfide, and pH were measured *in situ* during peak photosynthesis (12:00 Noon–2:00 PM) and at the end of the dark period (4:00 AM–6:00 AM) in approximately 30–40 cm-deep water. Needle microelectrodes (Unisense, Denmark) were mounted to a hand-driven micromanipulator and profiles recorded at 250-μm increments using a Microsensor Multimeter (Unisense, Denmark). Profiles of each constituent were measured in triplicate.

Small pieces of the microbialite mat (surface ~10 mm) were collected with stainless steel core liners (12–mm diameter) or by a knife near/at the location of microelectrode measurements. The pieces were transferred to a 15–mL Falcon tube and transported to the laboratory in the dark on ice. The total volume of select samples was determined by displacement of water. These samples were blotted dry, and chlorophyll was extracted in 5 mL methanol overnight at 4°C. Samples were then centrifuged (20 min at 3,000×*g*) and the extinction of supernatant was measured at 665 nm in a spectrophotometer (Carey 60 UV–vis). Chlorophyll concentrations were calculated using an extinction coefficient of 74.5 g – 1^.^L cm^−1^ (Stal et al., 1984).

Three core samples, similar to those described above, were pooled and EPS was extracted according to del Buey et al. (2021). Microbialite samples were mixed with deionized water (1,1 v/v water and sediment) and filtered using 45-μm nitrocellulose. The filtrate was centrifuged in an Eppendorf 5,810 R centrifuge for 10 min at 4,000 rpm and 4°C. The supernatant was precipitated using three volumes of ice-cold ethanol (96%) per volume of filtrate during 24 h at 4°C. The precipitate was recovered by centrifugation (6,000 rpm for 15 min) and 0.2 mL of HCl was added to remove any carbonate. The precipitate was placed into a dialysis bag (10 kDa), and dialyzed three times against 1 mM of EDTA for 24 h each time at 4°C. For the final dialysis step, deionized water (>18 MΩ) was used, for 24 h at 4°C. Following, 0.25 mL aliquots were taken from the dialysis bags and analyzed for hexose sugar contents, a measure for total EPS using the phenol-sulfuric acid method using n-glucose as a standard (Dubois et al., 1951; Decho et al., 2005).

### 2.2. Microbialite sampling

Additional microbialite samples were collected from ~0.3–1 m water depth by either chiseling the indurated subsurface (i.e., “hand samples”) or by using a hollow drill bit, which provided cylindrical “plugs” of microbialite and subsurface platform limestone (i.e., “cores”). Additionally, sediment samples were also collected from unconsolidated sedimented shallow-water areas adjacent to the platform. These unconsolidated sediments were cored in 1.4-cm inner-diameter syringe cores. Macrophytes were generally avoided while sampling, if possible.

In some cases, materials were preserved in the field. Specifically, some microbialite hand samples (~5 × 5 cm) were incubated for ~12–15 h in 1 μM CellTracker™ Green CMFDA (5-chloromethylfluorescein diacetate, hereafter CTG; Thermo Fisher) and then preserved in 4% paraformaldehyde. CTG is a viability indicator because it relies on esterase activity; it has been used in studies of marine microbial eukaryotes (Bernhard and Bowser, 1996; Bernhard et al., 2003, 2012; First and Hollibaugh, 2010; Thibault de Chanvalon et al., 2015). Other samples were preserved for DNA (see below).

Upon collection, hand samples to be maintained in the laboratory (i.e., kept living) were placed in plastic containers filled with ambient lake-surface water (~0.5 m deep). Microbialite cores were placed in cut off syringes with an inner diameter of 1.4 cm, which was slightly larger than the drilled core material. To prevent core movement, a thin wooden rod was lodged between the core edge and syringe barrel. The bottom of these syringes was plugged with polyester aquarium filter fiber to allow liquid exchange. Once the sample was secured in the syringe barrel, it was placed in a lake-water-filled well-fitting plastic tube to maintain core/microbialite orientation. Further processing information appears below (Section 2.6).

### 2.3. Isolation of organisms

Hand samples submerged in lake waters were maintained at room temperature on the laboratory windowsill at Woods Hole Oceanographic Institution (WHOI) or the University of Connecticut (UConn), so they were exposed to natural light–dark cycling. Motile organisms were noted to emerge from the samples, moving about on the bottom of the container or in its water. Organisms of interest were imaged (reflected or transmitted light microscopy) and isolated for further analyses.

Samples incubated in CTG and preserved in paraformaldehyde in the field were gently disaggregated or teased apart to obtain entrained eukaryotic inhabitants. In these cases, materials were examined with an epifluorescence-equipped dissecting microscope with fluorescein optics (480-nm excitation, 520-nm emission), surveying for fluorescent organisms.

### 2.4. rDNA sequencing

Genomic DNA was extracted from individual targeted protist specimens using a Quick-DNA Microprep Kit (Zymo Research). Attempts to amplify the DNA with foraminifera specific PCR primers, s14F1 and sB (RibB; Pawlowski, 2000) failed to yield any PCR products. Subsequently, we used the universal eukaryote PCR primers, 42F and 1498R, with slight modifications: EUK-42F CTCAAAGATTAAGCCATGCA, EUK-1498 R CACCTACGGAAACCTTGTTA. DNA (2–4 μL) was amplified by PCR using Onetaq® 2x master mix with standard buffer (New England BioLabs), in a total 50–μL amplification reaction, with the following amplification conditions: 94°C for 2 min, 35X (94°C for 30 s, 58°C for 30 s, 68°C for 90 s), and a final elongation step at 68°C for 5 min. PCR products were visualized on 2% agarose gel and purified using the E.Z.N.A. Cycle Pure kit following the manufacturer’s instructions (Omega, BIO-TEK). The obtained SSU-rDNA gene sequences were not of good quality, having many unidentified nucleotides. However, the NCBI-blast results indicated that the common free-form reticulate protist had similar sequence identity to *Chlamydomyxa labyrinthuloides*. Therefore, we designed specific primers to amplify *C. labyrinthuloides* and closely related species using forward_GLF_SAR (ACGCTTCTATACTGTGAAACTGCGAAT) and reverse_GLF_SAR (TACGACTTCACCTTCCTCTAAATAATGA). The DNA was amplified following the same protocol as above. PCR products were visualized on a 2% agarose gel, purified, and sequenced. The sequences were aligned using MUSCLE 3.8.31 and edited with BioEdit version (7.0.4.1). Additional available sequences from Foraminifera, Synurophyceae, Chrysophyceae, *Gromia* and other Stramenopiles were downloaded from the NCBI database and included in our alignment. Phylogenetic reconstructions were performed with RAxML (raxmlGUI 2.0) using 100 bootstraps and the GAMMA model of rate heterogeneity with GTR substitution model (Edler et al., 2021). The phylogenetic tree was visualized and edited using Figtree (v1.1.4; Rambaut, 2012).

### 2.5. Fine-scale distributions and microbialite fabrics

On three sampling occasions (May 2017, September 2017, November 2019), a series of microbialite cores was processed for sub-millimeter life-position determinations using the Fluorescently Labeled Embedded Coring method (FLEC; Bernhard and Bowser, 1996; Bernhard et al., 2003). FLEC cores were not collected on each trip as their preparation and analyses are extremely time consuming (i.e., beyond available resources). As noted above, these cores were secured on site in cut-off syringes, placed into appropriate tubes, and gently covered with lake water. CTG was introduced to a final concentration of 1 μM and the cores were maintained at ambient temperature and lighting (light–dark cycle). After approximately 30 h of CTG incubation, the cores were preserved in either 4% TEM-glutaraldehyde in 0.1 M sodium cacodylate (May 2017) or 4% paraformaldehyde (September 2017, November 2019). The May 2017 and November 2019 cores were preserved in the afternoon (during daylight), while the September 2017 cores were preserved soon after the dark period ended.

Cores were processed using our standard FLEC protocol (Bernhard et al., 2003) and were ultimately embedded in Spurrs’ or LR White resin. Each polymerized core was cut into ~0.6-mm thick sections, perpendicular to the microbialite-water or sediment–water interface, using a slow speed rock saw. Sections were examined via a Leica FLIII stereomicroscope with epifluorescence capabilities (480-nm excitation; 520-nm emission). Selected sections were examined and imaged with an Olympus FluoView 300 confocal laser scanning microscope (CLSM). All reflected-light and CLSM images are presented in vertical orientation, as *in situ*.

### 2.6. Microfabric disruption experiment

Approximately 4 months prior to the May 2017 collections, we obtained a culture of the freshwater foraminifer, *Haplomyxa saranae* (Dellinger et al., 2014). The culture was maintained in Volvic™ spring water at room temperature on the laboratory windowsill and exposed to the natural light–dark cycle of Woods Hole, MA, United States. The culture was offered two types of algae: *Chlorogonium elongatum* and *Chlamydomonas reinhardtii*. Only specimens that had deployed reticulopodia were used in our disruption experiment. Harvesting of specimens for use in the disruption experiment, which is described below, disturbed reticulopodia, but specimens are known to survive such transfer. The *H. saranae* culture was not prolific, and sometimes crashed, under our culture conditions so the experiment was only performed once.

In the laboratory, short cores (*n* = 6) were obtained by drill-press-assisted subsampling of hand samples of the GL microbialites and underlying platform carbonates collected in May 2017. These were placed into very short barrels of a 10-cc syringe (1.4–mm diameter), rubber stoppered at the bottom and filled with lake water. Prior to filling, the top of five core barrels were marked via notches to later identify core number. Three of the core barrels contained unaltered lake water while the other three contained lake waters with 1 mM colchicine (final concentration), which is a microtubule inhibitor (Bernhard et al., 2013). A second rubber stopper was firmly secured into the top of each syringe barrel and tested for leakage.

These microbialite samples were imaged with a Bruker Skyscan 1,272 Micro-CT; such scans were considered T_0_ or “before.” Each core was then “seeded” with 5 individuals of the cultured *H. saranae*, which were gently introduced to the microbialite surface via capillary tube. Each suite of three cores was placed, uncapped, in a transparent container along with appropriate lake water (±colchicine) to help prevent evaporation. These two containers were placed in a humid chamber (deionized water) near the laboratory windowsill to ensure samples were exposed to day-night cycles. Water levels were checked periodically and replaced as needed. After 3.5 months, the experiment was terminated by recapping the cores with rubber stoppers and scanning them again with micro-CT. These scans served as T_end_ or “after.”

### 2.7. Micro-CT scanning and alignments

Micro-computed tomography (micro-CT) scans of the core samples were performed at WHOI using a tabletop Skyscan 1,272 by Bruker. Transmitted radiographs were collected at 0.40° steps over 180°. The source voltage and current were set at 80 kV and 125 μA, respectively. An Al metal filter (1-mm thickness) was used to minimize low energy x-rays produced by the polychromatic source and reduce potential beam hardening artifacts. Pixel resolution ranged from 4.75–5 μm and scan duration was approximately 4 h. Raw radiographs were reconstructed into cross-sections using Bruker NRecon software. Thermal misalignment correction, beam hardening artifact correction, ring artifact correction, and limited smoothing were applied to the data to produce corrected 3-D volumes.

Corresponding dataset pairs (before and after experiment) were re-oriented using Dataviewer software so that XY, XZ, and YZ views were roughly similar. Then, they were uploaded together in the Avizo© software. Exact XYZ realignment of the corresponding datasets was achieved by thresholding the material volume in each dataset and applying a registration wizard tool to the thresholded volumes. Volume and shape variations could then be assessed by superimposing the corresponding volumes displayed in the same XYZ orientation. In addition to a visual assessment of the changes, the degree of anisotropy in each sample was obtained using the morphometry tool in Avizo©. The degree of anisotropy (DA) can vary from 0 to 1 and reflects how highly oriented substructures are within a defined sub-volume. This function allows for fitting ellipsoids within the material volume, which are used to generate material anisotropy tensors where eigenvalues relate to the length of ellipsoid axes. The DA value (1-long axis eigenvalue/short axis eigenvalue) is 0 for isotropic material and 1 for anisotropic material. The DA was used to quantify whether layering was created or disrupted during experiments. In each corresponding set of scans, DA values were compared for the entire volume (including indurated subsurface; “whole sample”), for 3–4 sub-volumes where layering was apparent (top ~5–7 mm; “whole microbialite layer”), and for 3–4 sub-volumes where disruption was most likely to have occurred (top 1–2 mm; “uppermost microbialite layer”). The uppermost microbialite layer is a subset of the whole microbialite layer. As corresponding sub-volumes had been precisely re-aligned, the same sub-volume boxes were used for direct comparison of DA values in corresponding datasets.

## 3. Results

### 3.1. Lake and platform biogeochemistry

Physicochemical characterization of lake waters overlying the platform showed a similar pattern each year (2017–2019) with a pH from 7.7–7.9 (April/May) to 7.5–7.6 (July/August) to 7.9–8.2 (October/November; 2018 data; see Table 1). This pattern, with a minimum in July/August, was mirrored in alkalinity, which decreased from 236–254 mg HCO_3_^−^ L^−1^ in April/May to 179–192 mg HCO_3_^−^ L^−1^ in July/August and increased to 224–232 mg HCO_3_^−^ L^−1^ in October/November. Similar summer minima existed for Ca^2+^ concentrations, which decreased from 9.8–10.2 mM in the spring to 8.9–9.1 mM in the summer and increased again to 9.2–9.4 mM in the autumn.

**Table 1:** Physicochemical conditions at southern end of the carbonate platform at Dead Man’s Point, Green Lake during three sampling events in 2018.

	Temperature (^o^C)	pH	Ca^2+^ Conc. (mM)	Alkalinity (mg.L^−1^ HCO_3-_)	Conductivity (μS.cm^−1^)	PAR (μE.m^−2^ s^−1^)	Chlorophyll *a* (μg.cm^−3^)
April 20, 2018	11.1	7.74	9.78	236	1707	1639	384
July 31, 2018	28.2	7.62	8.90	179	2492	1846	579
October 06, 2018	17.6	7.87	9.43	224	1780	1751	608

Data for other years is similar. Values are means of triplicate samples were taken during early afternoon at the site of microelectrode profiling, when additional samples were collected. Temperature, pH, (Ca^2+^) were measured in water column ~10 cm above microelectrode profiling site. PAR = photosynthetically active radiation (average of seven values, collected during profiling, taken 5 cm above platform). Chlorophyll *a* concentration was measured in surface 0.8–1.2 cm of the microbialite.

The corresponding temperature was 9.6–13.1°C (April/May), 20.8–28.2°C (July/August), and 10.1–17.6°C (October/November). The conductivity increased with increasing water temperature from 1707 μS.cm^−1^ at 11°C (April 2018) to 2,492 μS.cm^−1^ at 28°C (July 2018) before decreasing to 1780 μS.cm^−1^ at 18°C (October 2018). Light intensity, as measured by PAR, also had maximum values in July. The chlorophyll *a* concentration increased by 50–60% during the growth season, from 384 μg^.^cm^−3^ in the spring to 579 μg.cm^−3^ in the summer and peaked to 608 μg.cm^−3^ in the autumn. EPS, as expressed as hexose equivalents, increased from 105 μg.cm^−3^ in April 2018, to 379 μg^.^cm^−3^ in July 2018, thereafter decreasing to 237 μg^.^cm^−3^ in October, 2018.

**Figure 2:**
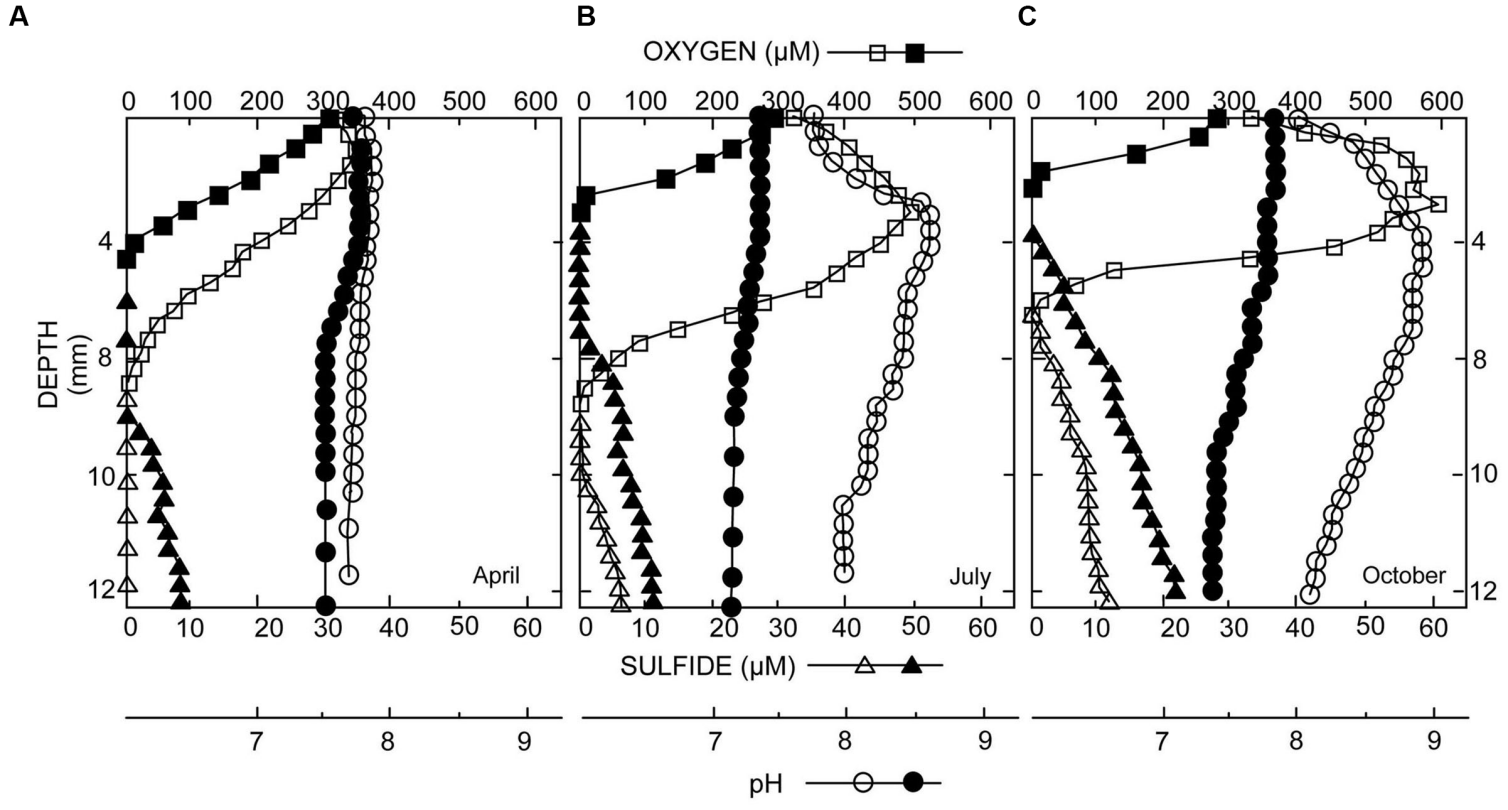
Microelectrode profiles of oxygen (O_2_), sulfide (H_2_S), and pH taken in microbialites from spring to autumn: **(A)** May; **(B)** July; **(C)** October. Open symbols were collected during daylight; closed symbols at the end of darkness. Peak values of O_2_ and H_2_S increase with time, which is indicative of mat development.

In DMP microbialites, depth profiles of oxygen and sulfide showed a seasonal pattern, with a minor daytime oxygen peak at ~1.5–2.5 mm, corresponding to the “uppermost microbialite layer, in spring to ~170% oxygen saturation in early summer and ~ 200% saturation in autumn (Figure 2). Hydrogen sulfide was only detected in summer and fall, at depth (i.e., >9 mm) during daytime and nearer the surface (~4-mm depth, whole microbialite layer) at end of night (Figure 2). In the spring, the pH profiles showed little change with depth. In the summer and autumn, pH maxima roughly coincided with the oxygen peaks, reaching values of 8.6 in the summer and 8.9 in the fall. Nighttime pH profiles typically showed a slight decrease with depth, which was more pronounced later in the season.

### 3.2. Platform microbialite appearance

**Figure 3:**
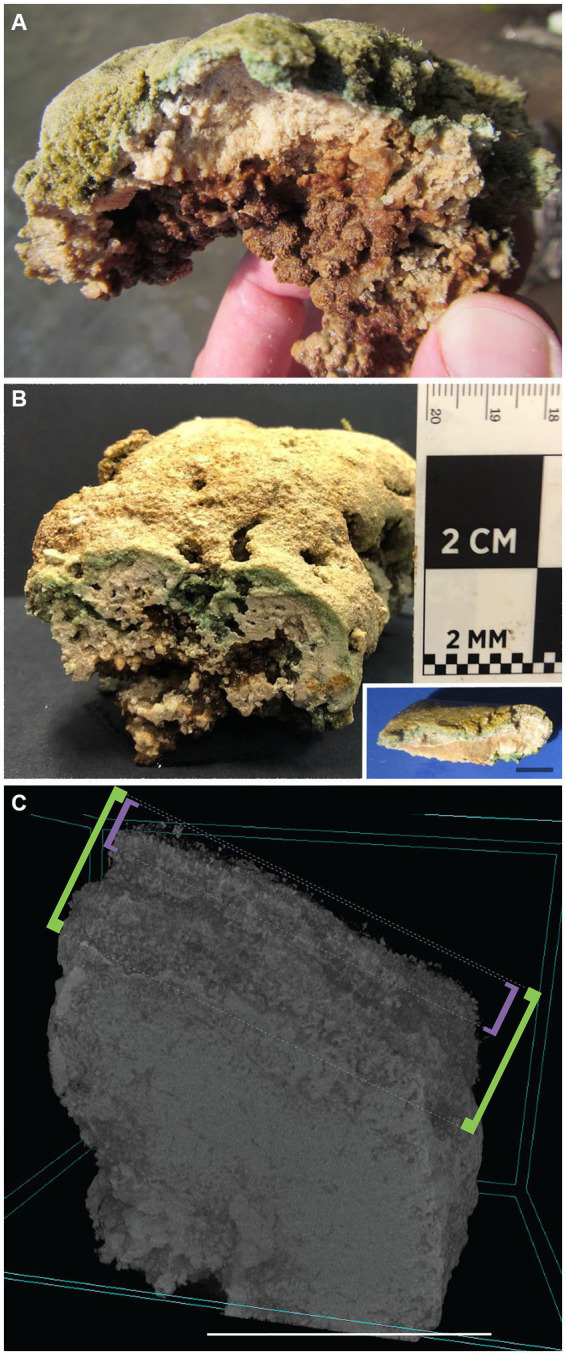
GL microbialites, collected May 2017. **(A,B)** Hand-samples showing layered structure at surface and clotted thrombolitic structure at depth. (Inset in **B**) Layered stromatolitic surface structure. **(C)** Micro-CT scan of a GL DMP microbialite core, showing “uppermost layer” surface (approximate 1–2 mm signified by purple brackets), “whole layer” (approximate 4–5 mm signified by green brackets), and more massive subsurface. Scales: Inset **(B)**, **C** = 1 cm.

While there could be patches of less structured deposits (e.g., some areas in Figure 1C) on the DMP, generally, visual inspection of the uppermost layer (top few mm) of the platform microbialites appeared laminated, while the near-surface (~3–6 mm) fabric appeared clotted (i.e., thrombolitic; Figures 3A,B) or massive. Voids of varied sizes (mm to cm) also occurred, typically— but not exclusively— beneath the overlying mat (Figure 3B). Higher magnification examination via micro-CT scanning revealed that layering can be clear or indistinct, depending on sample, while the indurated subsurface had no obvious layering and could be clotted or massive (Figure 3C). Early in the season (April/May), unconsolidated carbonate sediment was noted on the platform surface (Supplementary Figure S1A). Slightly denser flocculent material with a caramel color (presumably exopolymeric substances; EPS; Visscher et al. in prep) developed over time; it remained in patches on the platform surface, appearing to be most developed in FLEC materials collected in late summer (September; Supplementary Figures S2A,B). Mat layers in summer and autumn were typically dark green. Sometimes, what appeared to be carbonate precipitation within the mats was noted, especially in late summer or November (Supplementary Figure S2C). Microbialites collected in November had less EPS (i.e., October EPS data, above) but relatively thick unconsolidated sediments overlying the well layered microbialites (Supplementary Figures S2C,D).

### 3.3. Dead-Man platform benthos

#### 3.3.1. Metazoa

In “live” microbialite material (i.e., not preserved), ostracods occurred haphazardly. When chiseled hand samples were maintained in the laboratory for an extended time (>1 month), an ostracod “bloom” sometimes occurred. Our preserved CTG-labeled bulk microbialite samples yielded occasional metazoans, including ostracods, other crustaceans, and nematodes.

#### 3.3.2. Photosynthetic organisms

Regarding photosynthetic eukaryotes (based on frustule morphology and pigment presence), pennate diatoms were abundant in bulk “live” samples of both microbialite and off-platform sediments; aquatic plants of unknown identity were sporadically present in microbialite samples but never in our sediment samples. Microscopic observations showed both coccoid and filametous morphotypes in the surface microbialite mat, confirming prior observations of the DMP microbialite microbial community, where the dominant coccoid cyanobaterial form is a *Synechococcus* and the dominant filamentous cyanobacterial form is a type of Oscillatoracean (Thompson et al., 1990). The relative abundance of the filamentous cyanobacteria increased during the growth season from April/May to September/October (Murphy, 2021).

#### 3.3.3. Microbial eukaryotes

**Figure 4:**
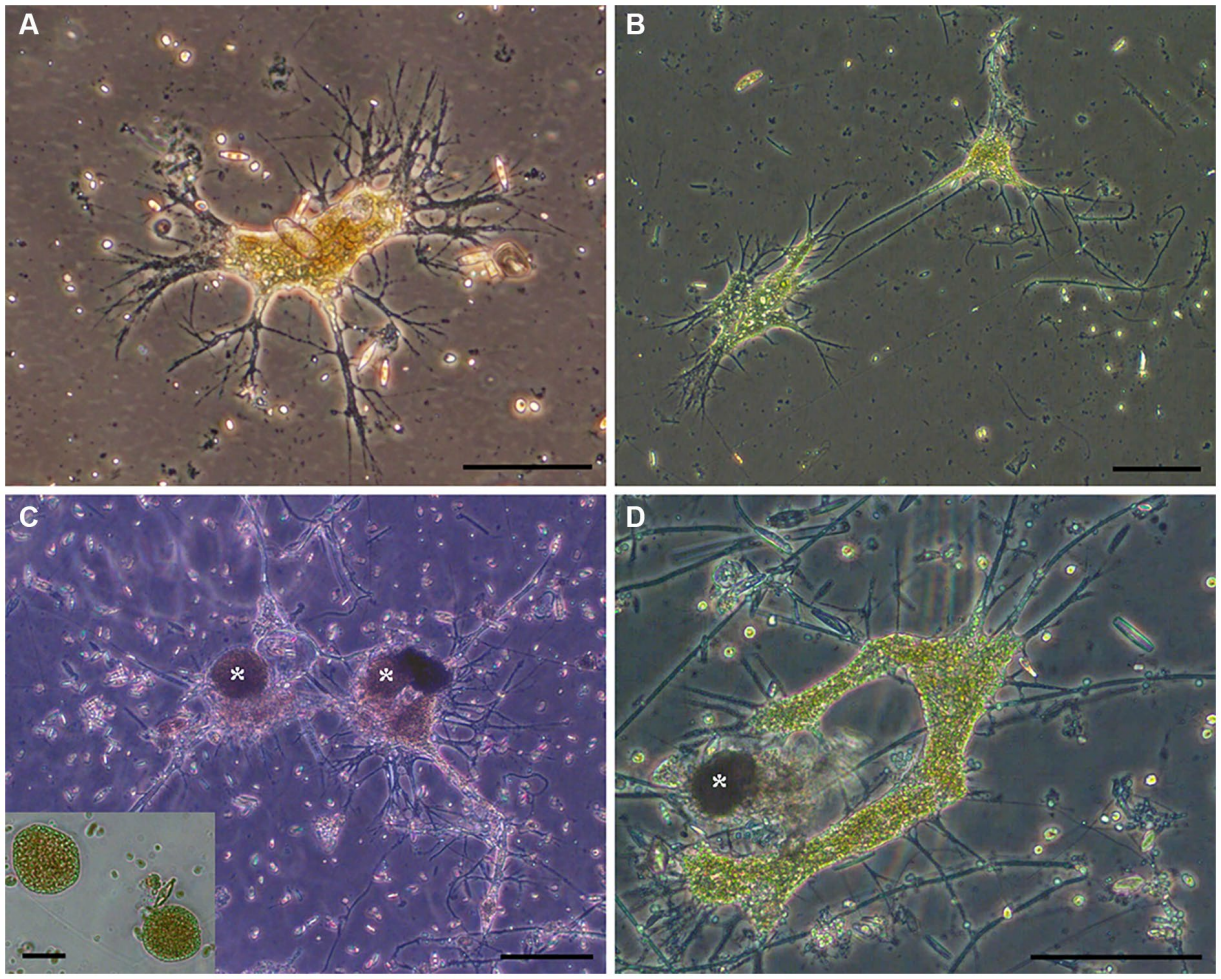
**(A–D)** Transmitted light micrographs of GL free-form reticulate protist (FFRP). Note anastomosing reticulopods emanating from free-form cell body. Collected in May 2017 **(A)**, August 2017 **(B,C)**, and September 2017 **(D)**. Cysts (*; inset) were often noted. Scales: **A** = 75 μm; **B,C** = 100 μm; **D**, Inset = 50 μm.

Aside from pennate diatoms, one microeukaryote morphotype was often noted in live microbialite material: a free-form “naked” cell body lacking a mineralized or thick organic covering with what appeared to be anastomosing pseudopods (i.e., reticulopods; Figure 4). This free-form microeukaryote, hereafter referred to as the “free-form reticulate protist” or “FFRP,” was typically pigmented greenish-brown to yellowish-brown, and generally did not exceed 250 μm in maximum cell-body dimension. In Petri dishes, these specimens were noted to be active, moving their reticulopodia as well as their cell bodies (plasmodium), as evidenced in seconds- to minutes-scale time-lapse compilations (Supplementary Video S1). For example, over 14 min, one specimen moved laterally nearly 500 μm. The reticulopodia could extend hundreds of microns when specimens occurred on flat surfaces. For specimens with a relatively compact cell body, pseudopodial extension distances typically exceeded 1.5 times the plasmodium diameter, although sometimes extension reached over three times the cell-body diameter. The extent of their reticulopodal extension *in situ* is not known.

Some ovoid/spheroid entities noted in fresh GL materials were isolated indvidually into clean Petri dishes with GL waters (Figures 4C,D). These spheriods typically appeared vacuolated (Figure 4C inset). Upon examination the next day, the spheroids were absent but a free-form individual with reticulopodia was present; these were identical in appearance to the GL free-form reticulate protist. Henceforth, we refer to these ovoid /spheroid entities as “cysts.”

Individuals of the FFRP were present during each collection trip (if investigated for such; Table 2). The amorphous morphotype occurred in microbialites and platform sediments but never in the water column. In general, fewer FFRP specimens occurred in non-platform sediments compared to microbialite samples. Quantification of microbialite samples was unrealistic mainly due to difficulty in obtaining undisturbed samples of consistent volume (rock/microbialite rubble) and because preserved specimens were not identifiable due to lack of robust theca, resulting in less-than-ideal preservation.

**Table 2:** Qualitative survey of the free-form reticulate protist morphotype in different GL habitats, over time.

	05/17	8/17	9/17	4/18	5/18
Water column	A	A	A	A	A
EPS “fluff”	C	C	C	N/A	C
Uppermost microbialite	C	C	C	C	C
Near-surface microbialite	N/A	P	P	C	C
Sediment	C	C	P	N/A	N/A

A, absent; P, present; C, common; N/A, not available. EPS “fluff” includes entrained sediment grains, uppermost microbialite layer = top ~3 mm; Near-surface microbialite = ~3–10 mm; Sediment = off-platform unconsolidated sediments.

### 3.4. Free-form reticulate protist taxonomic affinities

**Figure 5:**
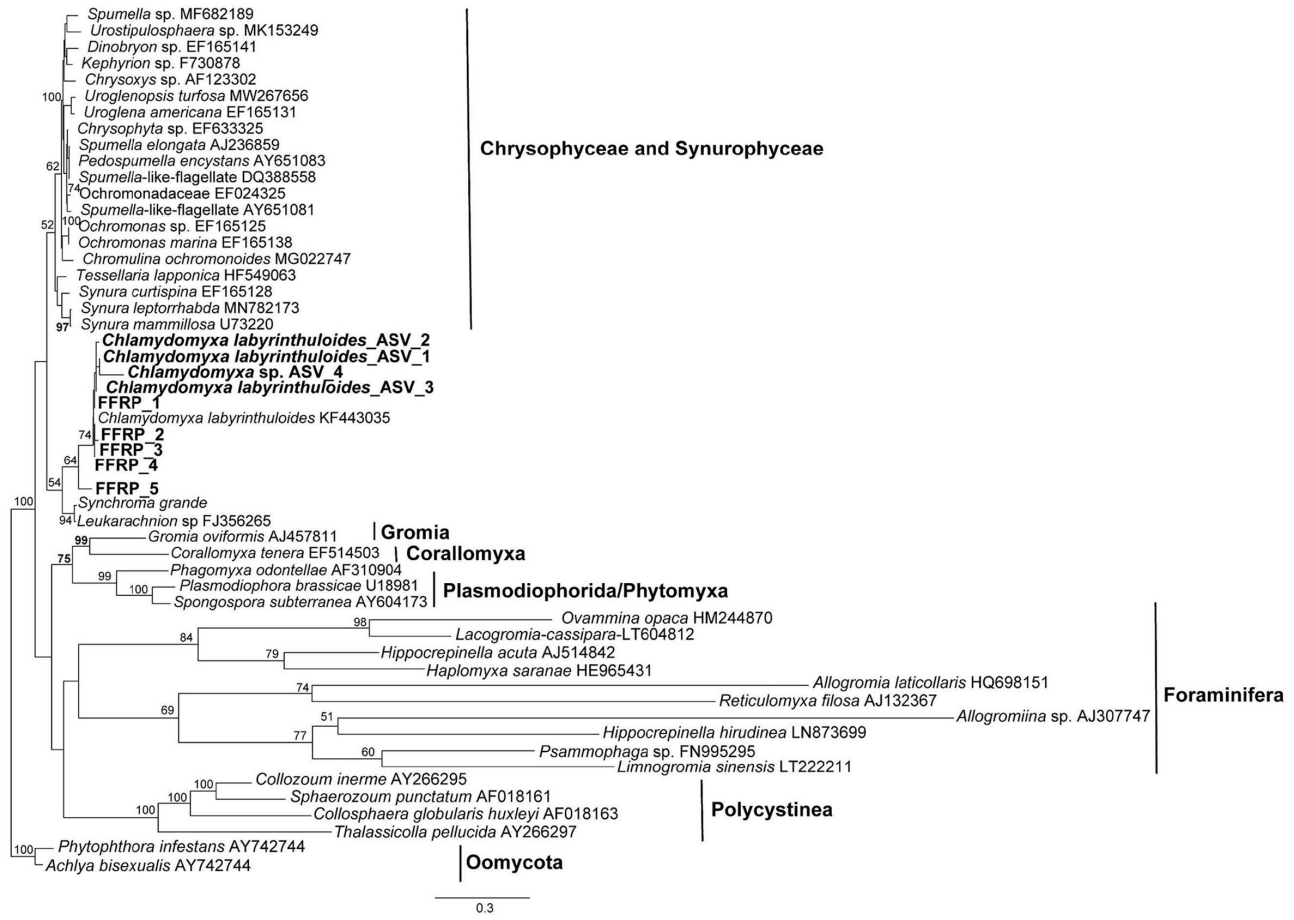
Maximum-likelihood phylogenetic analysis, showing the placement of the GL free-form reticulate protist (FFRP), showing it is closely related to *Chlamydomyxa labyrinthuloides* in a tree consisting of representatives of Synurophyceae and Chrysophyceae, Foraminifera, *Gromia*, *Corallomyxa*, Phytomyxea, and Polycistina. Posterior bootstrap value support greater than 50% are shown at the nodes; tree rooted by Oomycota.

Sequences of the free-form reticulate protists that were isolated from DMP microbialites are deposited in GenBank under Accession Numbers OQ786765-OQ786769. A phylogenetic tree (Figure 5) showed that the GL FFRP clusters with *C. labyrinthuloides* and both form a supported subclade with *Leukarachnion* sp. (Grant et al., 2009) and *Synchroma grande* (Horn et al., 2007), which are Stramenopiles.

eDNA sequences that were obtained for both 16S and 18S using MiSeq had ASVs assigned to *C. labyrinthuloides*; these are deposited in GenBank under Accession Numbers OQ786770-OQ786773. These were similar to sequences obtained from direct PCR amplification of rDNA using species-specific primers (above). These *C. labyrinthuloides* eDNA sequences occurred in three of 17 samples (Table 3), in April and October. eDNA sequences of an unidentified species of *Chlamydomyxa* was detected more commonly and more abundantly, being noted in five of 17 samples, exclusively in July and October. All *Chlamydomyxa* were noted in microbialite samples, but never in off-platform sediments, “fluff” (unconsolidated sediment / EPS), or overlying waters (Table 3).

**Table 3:** ASV abundances/number of reads for *C. labyrinthuloides* and unidentified *Chlamydomyxa* sp. sequences based on eDNA analysis, by 2018 month of sampling (Mat = microbialite; Fluff = EPS + unconsolidated materials overlying microbialite).

	Spring			Summer			Autumn					
	April			July			October					
Taxa	Mat 1	Mat 2	Mat 3	Mat 6	Mat 7	Mat 5	Mat 7	Mat 6	Mat 5	Fluff 1	Fluff 2	Fluff 3
*C. labyrinthuloides* (KF443035.1.1713)	11	0	0	0	0	0	0	0	0	0	0	0
*C. labyrinthuloides* (KF443035.1.1713)	0	0	0	0	0	0	0	0	6	0	0	0
*C. labyrinthuloides* (KF443035.1.1713)	0	0	0	0	0	0	2	0	0	0	0	0
*Chlamydomyxa* (unidentified sp.)	0	0	0	47	16	0	19	19	14	0	0	0

Sequences were absent from October’s two water samples and three off-platform sediment samples (not tabulated).

### 3.5. Sub-millimeter distributions

#### 3.5.1. Fabric preservation

**Figure 6:**
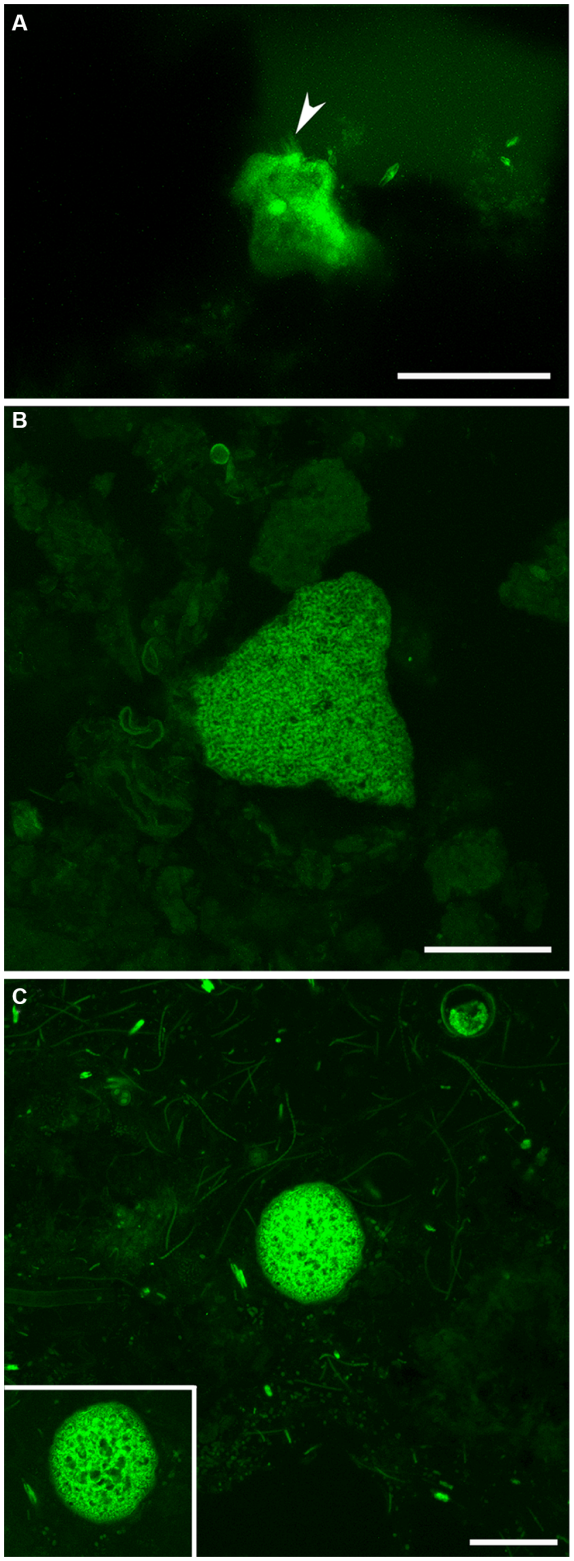
LSCM images (z-stacks) showing fluorescently labeled GL FFRP in their life positions in FLEC preparations. **(A)** Lobose free-form individual, in the surface 1 mm, with faint pseudopodial trunks extending upward from the main body (arrowhead). Three pennate diatoms are also visible in this off-platform sediment core. **(B)** Free-form specimen at 4 mm depth, collected from off-platform sediments. **(C)** Spherical vacuolated cyst-like form from the surface 1 mm of microbialite, among coccoid and filamentous microbes. Inset. Single-slice image of same cyst, showing vacuolization. Number of images compiled/distance between images (μm): **A** = 63/0.5; **B** = 37/0.5; **C** = 31/0.4; inset = 1. Scales: **A,B** = 100 μm; **C** = 50 μm. **A** collected in May 2017; **B** in November 2019; **C** in September 2017.

**Figure 7:**
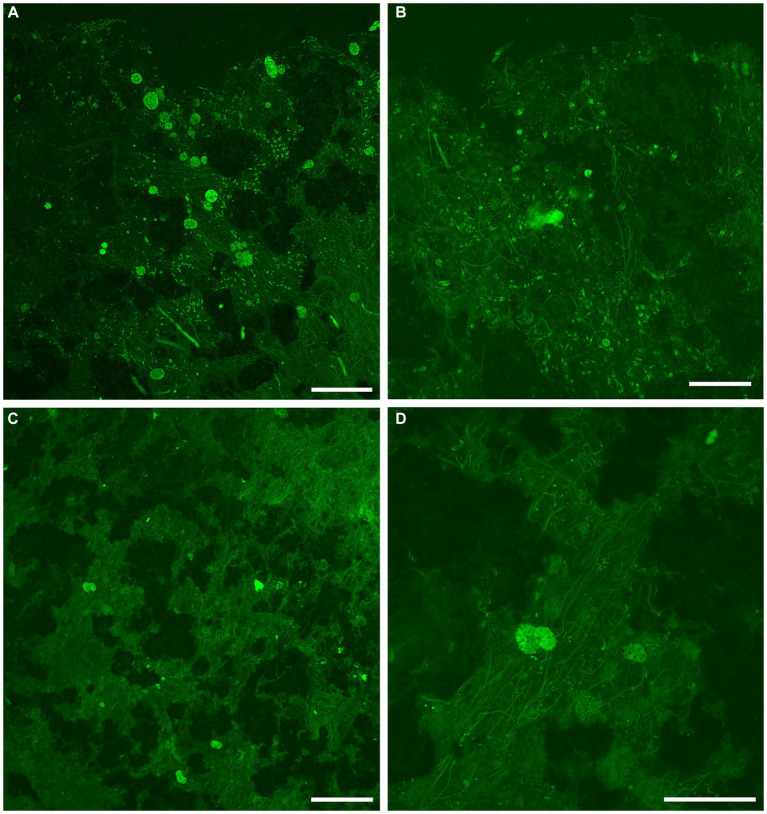
Overview LSCM images (z-stacks) showing fabric and microbiota in GL microbialites collected in May (FLEC). Black and/or dark green is sediment, rock, and/or pore/bottom waters; dim to moderate green is EPS or decaying organics. **(A)** Abundant cyst-like specimens in clotted fabric at microbialite surface. **(B)** Individual FFRP near microbialite surface (~0.5 mm deep), with variety of microbial morphotypes including pennate diatoms. **(C)** Zone of microbialite ~3-mm deep with many filamentous microbes in addition to 3 putative FFRP and what may be two vacuolated cysts. **(D)** Higher magnification view of portion of **C** showing two superimposed larger vacuolated organisms, inferred to be FFRP cysts, among filaments. Number of images compiled/distance between images (μm): **A** = 55/1.4; **B** = 71/0.7; **C** = 17/1.4; **D** = 59/0.7. Scales: **A,C** = 200 μm; **B,D** = 100 μm.

**Figure 8:**
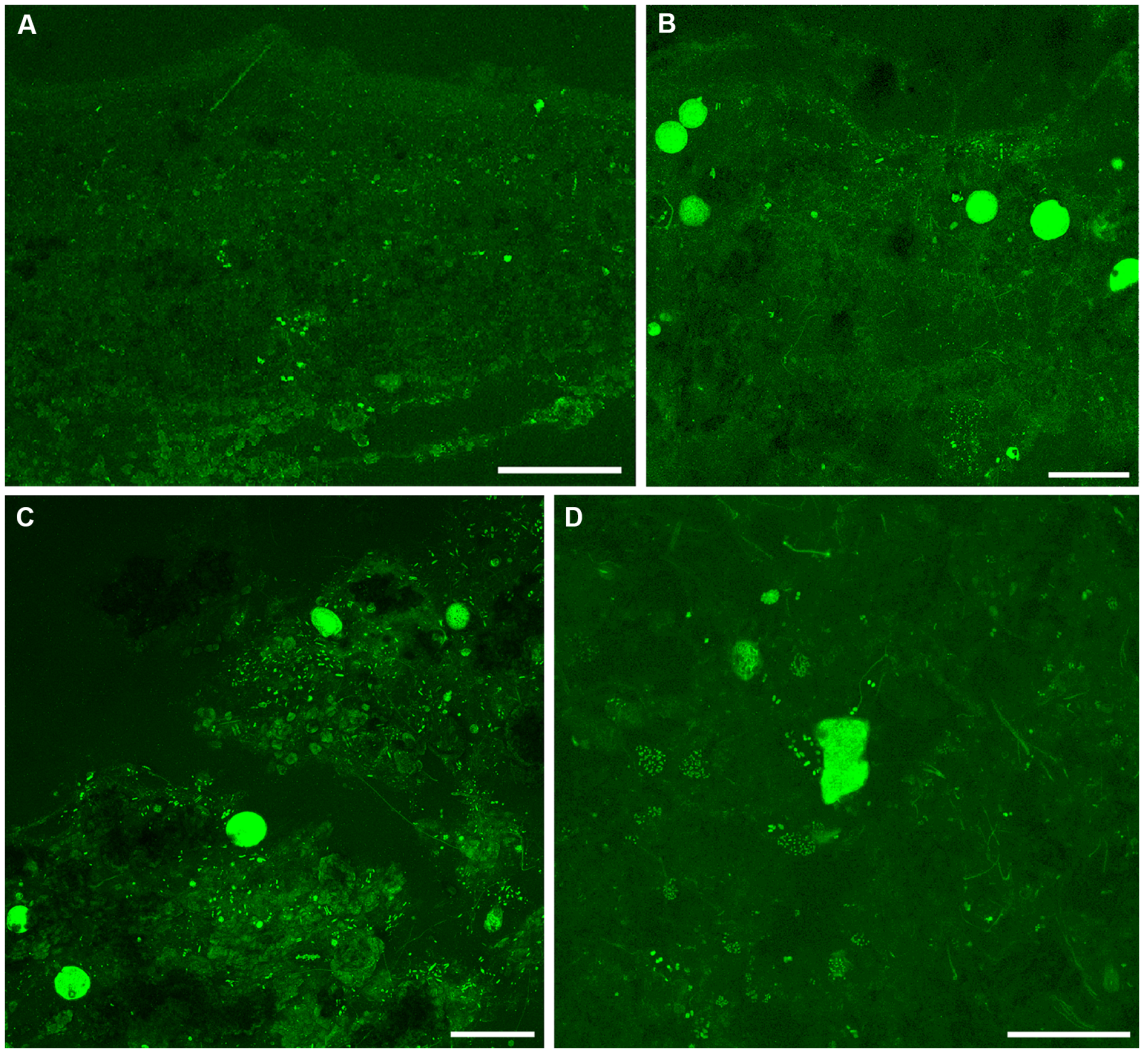
Overview CLSM images (z-stacks) of fluorescently labeled microbes in GL microbialites, collected in September 2017 (FLEC). **(A)** Slightly layered fabric at the microbialite surface, overlying a layer of relatively larger blocky grains with moderate autofluoresce. **(B,C)** Images showing abundance of cyst-like specimens in clotted fabrics, within the surface 1 mm, with mostly filaments **(B)** and mostly pennate diatoms **(C)**. **(D)** A free-form specimen from 9 mm depth, among possible flagellated cells and aggregates of coccoid microbes, with some filamentous forms. Number of images compiled/distance between images (μm): **A** = 28/1.4; **B** = 48/1.4; **C** = 95/1.4; **D** = 33/0.7. Scales: **A–C** = 200 μm; **D** = 100 μm.

**Figure 9:**
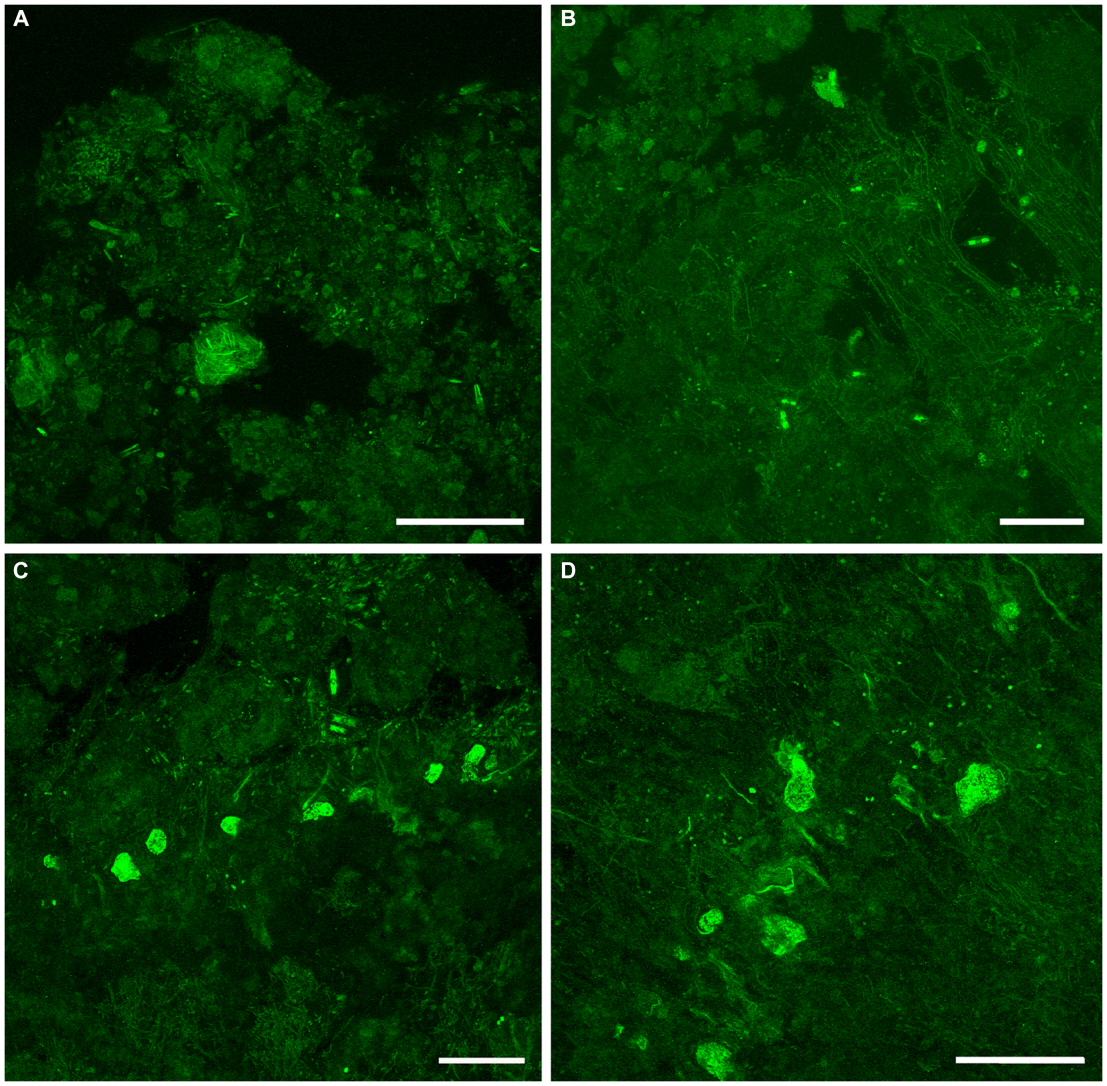
Overview CLSM images (z-stacks) of fluorescently labeled microbes in GL microbialites, collected in November 2019 (FLEC). **(A)** Lobose specimen near microbialite surface (~0.2 mm deep), in pore-water void, among clumped EPS. **(B)** Free-form specimen in pore-water void within surface mm, among thin filaments and EPS or remnant organics. **(C)** Six putative FFRP in lobose form at ~2 mm depth, among pennate diatoms and filaments. Note dominance of filaments below lobose specimens and diatoms above. **(D)** Five lobose vacuolated individuals among varied microbes, ~2 mm depth. Number of images compiled/distance between images (μm): **A** = 44/0.5; **B** = 43/0.7; **C** = 31/0.7; **D** = 41/0.5; Scales: **A–D** = 100 μm.

Examination with reflected light, epifluorescence, and CLSM revealed that FLEC sections of microbialite cores with layering (i.e., stromatolitic) maintained their fabric (i.e., layers) through the FLEC processing (Supplementary Figures S1, S2C,D). This indicated that little, if any, sub-millimeter-scale disturbance occurred during the FLEC procedure, similar to prior studies (Bernhard et al., 2003, 2013, 2023). As usual for FLEC, the resin imparted minor background autofluorescence, helping to distinguish what was porewater versus sediment grains or indurated rock. The GL carbonate grains and EPS had moderate autofluorescence, causing detection of fluorescent microbes to be difficult at dissecting-microscope magnifications (not shown); the best visualizations were via confocal imaging. Brightly fluorescent (i.e., enzymatically active) organisms were present in all FLEC samples (Figures 6–9; Supplementary Figures S1, S3–S5), both from microbialites and off-platform sediments.

#### 3.5.2. Documentation of free-form reticulate protist-like specimens in life position

Examination of FLEC sections from microbialites and off-platform sediments revealed the presence of entities similar to the FFRP that was noted in “live samples” (compare Figures 4, 6A,B), as well as the cysts (compare Figures 4C,D, 6C). While well-developed reticulopodia were not observed in FLEC sections, we did observe faint evidence of reticulopodial trunks in some free-form specimens (Figures 6A, 7B). Typically, CLSM confirmed transmitted light observations that the FFRP cysts are typically vacuolated (Figures 6C, 7D, 8B,C). Rarely, non-vacuolated spherical specimens were noted (not shown); these were attributed to metazoan eggs and not further discussed.

#### 3.5.3. Distributions in microbialites over time

In May 2017 samples, the uppermost microbialite layer (surface ~2–3 mm) of FGL microbialites showed rather loose, faint layering (Figure 3C; Supplementary Figure S1) but could also appear leiolitic (no well-structured fabric; Figures 7A,B). The near-surface (>2 mm) microbialite fabric did not display any undisrupted strong layering in May (Figures 3C, 7C,D; Supplementary Figures S1A,B). The community was dominated by fine filamentous microbes, especially in the uppermost microbialite layers of FLEC samples (Figures 7C,D; Supplementary Figure S1C). Coccoid microbes were spatially abundant in some areas (Figure 7A) while diatoms also occurred near the sediment–water interface, although not abundantly. At this time, cells that appeared to be the FFRP were noted in FLEC materials (Figures 7B,C; Supplementary Figure S1C) and what could be FFRP cysts, due to vacuolization, were also noted (Figures 7A,C,D), sometimes in relatively high densities (Figure 7A) and as deep as 3 mm (Figures 7C,D). Other organisms noted in microbialites from May FLEC materials include nematodes (Supplementary Figure S3F), an unidentified crustacean (Supplementary Figure S3E), macrophytes (Supplementary Figures S3E, S5D) and a possible testate amoeba (Supplementary Figures 4C,D).

In September, microbialite surfaces sometimes appeared as faint layers (Figure 8A; Supplementary Figure S2A), but these undulating layers were due to the presence of considerable overlying “fluff,” considered to be EPS (Supplementary Figures S2A,B). The microbialite surface, below the fluff, in September was sometimes layered at the sub-mm scale (Figure 8B) but could also appear clotted (Figure 8C) while the subsurface was unstructured (Figure 8D). The September community was dominated by FFRP-like vacuolated cysts (Figures 8B,C) while few free-form specimens were observed (Figure 8D). The cysts were generally in the uppermost surface while one FFRP was noted at 9 mm depth (Figure 8D). An oblong vacuolated specimen in Figure 8C had a theca-like covering (Supplementary Figure S4A) similar to those of thecate amoebae. Possible flagellates were noted in a loose grouping (Supplementary Figure S5A). Filamentous microbes were not as common as in May, although aggregations of thicker filaments were observed at depth (Supplementary Figure S5B); coccoid groupings were noted in the lower layers of the microbialite (Figure 8D) and pennate diatoms could be relatively common in the uppermost layers (Figure 8C). Occasional macrophytes were noted in the September FLEC samples (Supplementary Figure S5C). Ostracods were noted in the microbialites in September (Supplementary Figure S3D).

In November, the uppermost ~2 mm of microbialite surfaces always appeared clotted, without linearity (Figure 9A; Supplementary Figures 2C,D); sub-mm-scale voids were relatively common (Figures 9A–C). The near-surface of microbialites (~2–4 mm) did appear, however, to show layering, evident in fabric (Supplementary Figures S2C,D) and as stratified microbial communities (Figures 9B–D). For example, what appeared to be numerous FFRP were aligned along a horizon (Figures 9C,D), roughly dividing filamentous microbial forms below and pennate diatoms (Figure 9C) or coccoid microbes (Figure 9D) above. The depth of these horizons was typically about 2 mm, although some dense aggregations of coccoid cells were noted at ~4–mm depth (Supplementary Figure S5D). In some cases, the RRFP appeared associated with a void within the microbialite (Figures 9A,B). Few spherical vacuolated cysts were noted in November FLEC samples; one highly elongated, vacuolated form was noted in a microbialite sample (Supplementary Figure S2B). The largest metazoans observed in our FLEC materials were noted in November sediment samples, where crab-like and tanaid-like specimens were documented (Supplementary Figures S3A,B, respectively). Ostracods were also observed in off-platform sediments in November (Supplementary Figure S3C).

### 3.6. Microbialite microfabric disruption experiment

**Figure 10:**
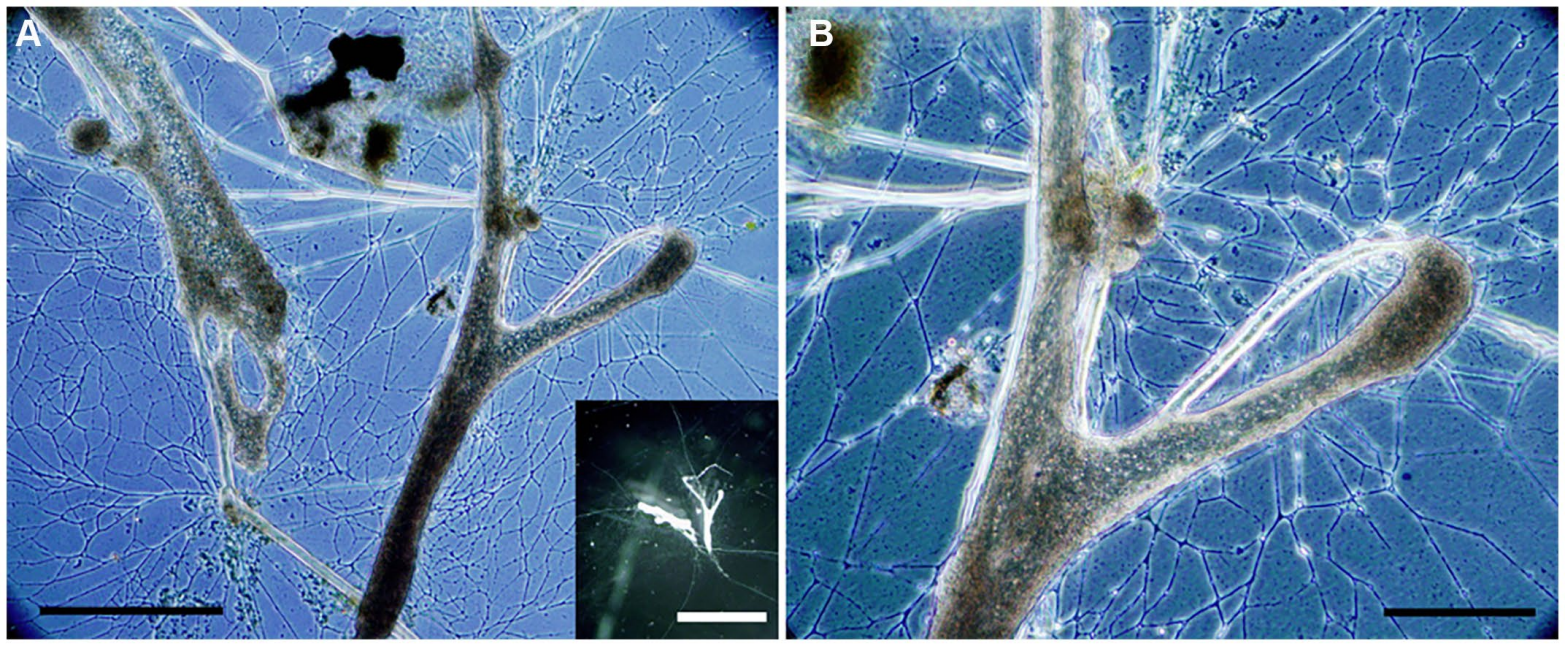
Transmitted light micrographs of *Haplomyxa saranae* in a Petri dish. **(A)** Two *H. saranae* showing deployed reticulopodia forming dense networks. Inset. Reflected light micrograph of same specimens showing partial extent of reticulopodial reach. **(B)** Higher magnification view of specimen in **A**, showing magnified reticulopods. Scales: **A** = 250 μm; **B** = 100 μm; Inset = 1 cm.

The cultured freshwater foraminifer *H. saranae* typically had extensive reticulopods (Figure 10) but would also encyst (Dellinger et al., 2014).

**Figure 11:**
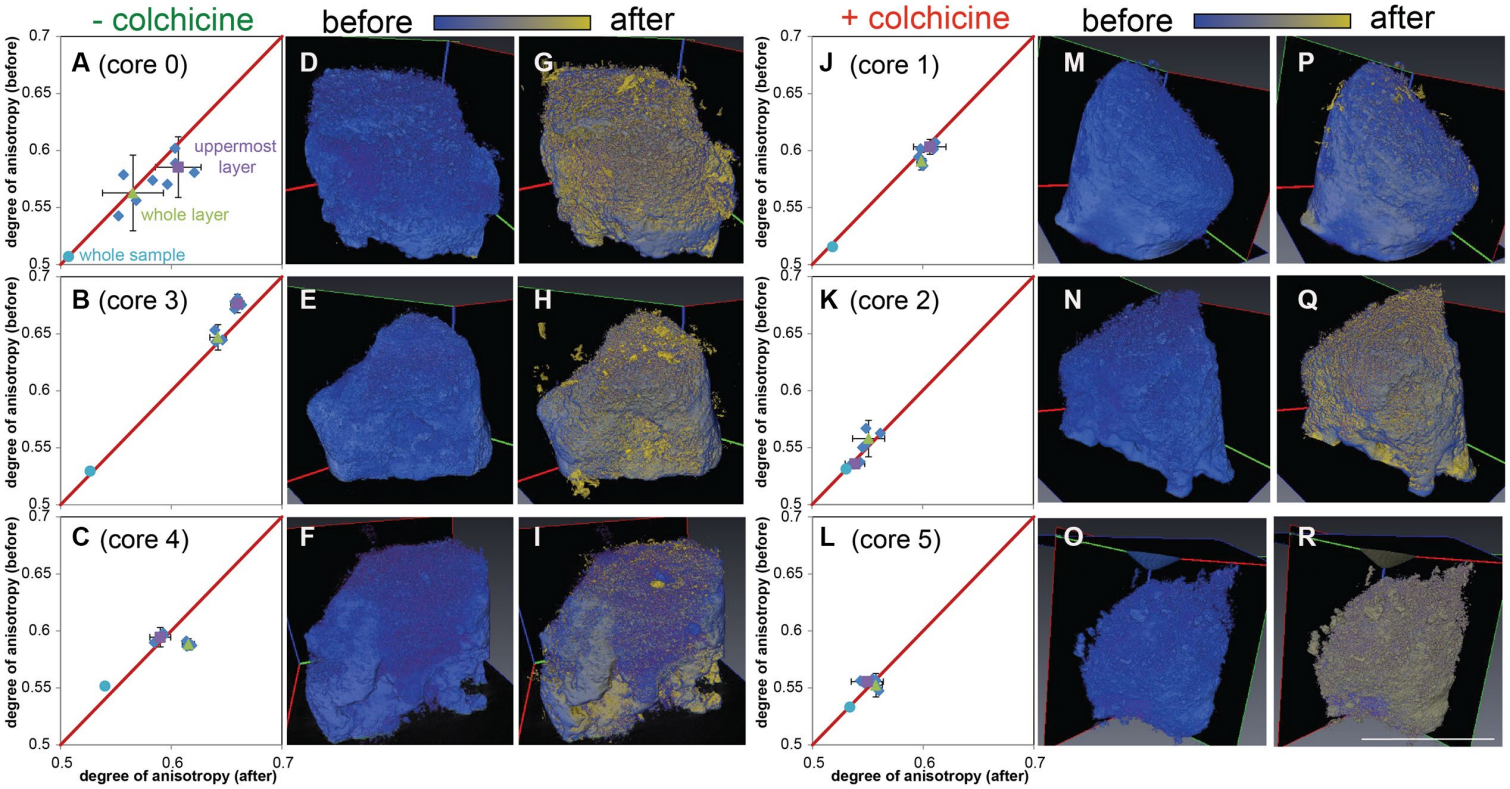
Micro-CT scans of cores from the *H. saranae* seeding experiment. Left side **(A–I)** shows cores incubated without inhibitor; right **(J–R)** shows cores with microtubule inhibitor. **(A–C,J–L)** Plots showing degrees of anisotopy of panels **G–I**,**P–R**, respectively. **(D–F,M–O)** T_0_ (before) scans, with initial volumes in blue. **(G–I,P–R)** Superimposed T_0_ and T_end_ micro-CT scans with initial volumes in blue and volumes after experiment in yellow. Gradient shades between blue and yellow reflect superimposition of the two colors and near-identical volumes in T_0_ and T_end_ micro-CT scans. Material growths during experiment are detected as new yellow material. Scale = 1 cm (same for all scans).

A comparison of the layered microbialite fabrics before (T_0_) and after (T_end_) the ~3.5-month microfabric disruption experiment showed modifications of the layers in cores incubated without colchicine (*n* = 3; Figures 11A–I; Supplementary Table S1), where reticulopodial activities were not arrested. In particular, micro-CT scans reveal that the top 1–2 mm of these cores (“uppermost layer”; Figure 3C) recorded different degrees of anisotropy in core 0 (Figure 11A) and core 3 (Figure 11B) at T_0_ and T_end_, consistent with microbialite fabric disruption in the uppermost layers of these samples during the experiment. Additionally, growth of new material was detected by superimposing T_0_ scans (blue volumes) with T_end_ scans (yellow volumes), as evidenced by new yellow voxels primarily localized around the layered portions of the sample (Figures 11G–H). The third core incubated without colchicine (core 4) also showed growth of new material during the experiment (yellow voxels; Figure 11I), but most of the microbialite fabric disruption may have occurred in deeper microbialite layers in this core, as evidenced by a change in anisotropy recorded in the “whole microbialite layer” (Figures 3C, 11C). Conversely, the micro-CT scans of the inhibited cores (*n* = 3; Figures 11J–R; Supplementary Table S1), where reticulopodial activities were arrested, showed no detectable changes in degree of anisotropy from T_0_ to T_end_ (Figures 11J–L). Additionally, the lack of microbialite fabric disruption in these cores is consistent with limited (Figures 11M,P) or non-detectable growth of new materials during experiment (Figures 11N,O,Q,R). The similar degree of anisotropy recorded in the” whole sample” in all experiment cores (likely volumetrically dominated by the non-layered portion of each sample) indicates that structural changes, when detected, were localized in the layered portions of the samples.

## 4. Discussion

### 4.1. Seasonality of lake geochemistry

Multi-year observations revealed seasonal fluctuations of physicochemical conditions in the water column overlying the microbialite platform, showing a consistent pattern of an increase in temperature, conductivity and light regimes from spring to summer, followed by a decrease from summer to fall. The pH, (Ca^2+^) and alkalinity followed a reverse trend, with the lowest values in the summer. Based on oxygen and sulfide microprofiles and chlorophyll *a* concentrations of microbialite layers, the photosynthetic microbial activity continued to increase from mid spring to late fall, and sharply decreased during the winter, when occasionally the platform was covered by ~10 cm of ice for several months (e.g., January – March 2018). Such trends in biogeochemistry are typical for temperate microbial mat ecosystems (Visscher et al., 2022).

It may be important to note that the surface few (2–4) mm of the microbialite (uppermost layer) experiences depleted oxygen concentrations, if not anoxia, at nighttime although concentrations of hydrogen sulfide remain low to undetectable shallower than 4 mm. The microbialites represent a dynamic chemocline, which may be a challenge to aerobic fauna.

The platform and microbial mats of GL are unique in that they develop in a “hardwater” lake setting that is permanently stratified. The oxic mixolimnion supports blooms of coccoid cyanobacteria, such as *Synechococcus* (Thompson et al., 1997; Cohen et al., 2023). These blooms produce whiting events, comprising fine-grained (~5-μm diameter) calcium carbonate minerals. Some of these mineral particles sink to the monimolimnion and form the bottom sediments of the lake, but a substantial part of the calcium carbonate settles on the surface of the DMP platform and is trapped and bound to the surface mat by EPS, the concetration of which increased first, then decreased near the end of the annual growth of the mat community. As in the Proterozoic ocean, the oxycline and anoxic bottom waters are characterized by vigorous sulfur cycling (Thompson et al., 1990; Zerkle et al., 2010; Cohen et al., 2023), and are possibly involved in delivery of essential nutrients to the surface waters, including to the DMP microbialites (Fry, 1986). Another unique feature of GL contributing to the formation of the extensive carbonate platform at DMP is the intrusion of calcium-rich groundwater (Figure 1A; Thompson et al., 1990), which elevates the saturation index of calcite and aragonite in the monimolimnion.

### 4.2. Microbialite structure over time

The DMP microbialites seemingly have an annual cycle, where the surface (uppermost layers) appeared, on the mm-scale, to have loosely formed laminations and sometimes a concreted leiolitic surface horizon in the spring. Copious EPS masked the mat surface in summer, while distinct layers, including green (presumably chlorophyll-rich) ones, were noted in the top ~4–5 mm of DMP microbialites in autumn, when crystallites, presumably of calcite (Brunskill, 1969), could also be observed in these layers.

### 4.3. Green Lake eukaryotes

Microbialites, especially stromatolites, are considered Earth’s earliest widespread ecosystem (Allwood et al., 2006; Knoll et al., 2016), one which evolved before the origin of metazoans and likely even before the origin of microbial eukaryotes. Today, eukaryotes are integral ecosystem partners with DMP microbialites. Our morphological and life position (FLEC) observations reveal that the platform microbialites host eukaryotes—most commonly protists (pennate diatoms, free-form reticulate protist), nematodes, and ostracods—plus the expected additional primary producers of cyanobacteria and macrophytes.

It may be somewhat surprising that relatively few ciliates and euglenozoan flagellates were noted in our GL FLEC materials because, in marine sediments, both can be commonly observed via FLEC (Bernhard et al., 2003, 2023) euglenoid flagellates can be highly abundant (Bernhard et al., 2000), sometimes as near monospecific “swarms” (Figure 7C; Bernhard et al., 2003). The apparent dearth of euglenozoan flagellates in our GL FLEC materials may be an artifact, since flagella are difficult to discern in FLEC materials and flagellates may be confused with coccoid cyanobacteria, as both types can be ~10 μm in diameter. Ciliates and flagellates were not observed in our live materials, but they were not a focus of our searches so they may have been overlooked.

### 4.4. GL free-form reticulate protist identification and phylogeny

The FFRP found in GL microbialite and sediment samples had similar morphology to that of the freshwater foraminifer *H. saranae* (Figure 10; Dellinger et al., 2014) and other “naked” foraminifers such as *Reticulomyxa filosa* (Glöckner et al., 2014) although *R. filosa* and *H. saranae* are considerably larger than the GL FFRP. The dimensions of the free form or amorphous fluorescent specimens in GL FLEC materials were consistent with the cell bodies of GL microbialite FFRP observed in Petri dishes.

Based on light microscopy, the GL free-form reticulate protist appeared to have anastomosing reticulopods, a characteristic of retarian protists (Foraminifera, Radiolaria; Bowser and Travis, 2002; Cavalier-Smith et al., 2018). Foraminiferal reticulopodia have an anastomosing network as well as substantial tensile strength (Bowser et al., 1992), enabling foraminifers to rend biofilms (Bernhard and Bowser, 1992) as well as impact marine stromatolite microfabric (Bernhard et al., 2013). The motility of the FFRP is similar to that of the freshwater “naked” foraminifer *H. saranae*. Whether or not the FFRP pseudopodia display other ultrastructural features of reticulopodia (Travis and Bowser, 1991; Bowser and Travis, 2002) awaits further study. While we know the GL FFRP appears to bear reticulopods, there was little evidence of reticulopods in FLEC materials but this is not surprising because reticulopodia typically are not well-preserved after fixation unless certain protocols are enacted (Bernhard et al., 2013) that were not used in this study.

Unlike their better known marine counterparts, freshwater foraminifers, e.g., *H. saranae,* undergo encystment (Dellinger et al., 2014; Wylezich et al., 2014). The laboratory-based observation that GL cyst-like spheroids emerged to become the FFRP bolstered our original inference that this GL morphotype was a freshwater foraminifer. Comparable dimensions of FLEC cysts and cysts observed in the laboratory along with the vacuolated nature of both entities further supported our original inference. According to our single-cell sequencing results, however, the Green Lake FFRP is closely related to *C. labyrinthuloides,* which is known to have finely branched, reticulated pseudopodia, yellowish-brown to greenish-brown pigmentation (Archer, 1875), be well vacuolated (vs. a single contractile vacuole), and an encystment stage called an aplanospore (Wenderoth et al., 1999). Environmental DNA sequences also grouped with known *C. labyrinthuloides*. While the GL FFRP clearly has reticulated pseudopodia, its sequence does not cluster with Rhizarians, which are known for their reticulopodia (Bowser and Travis, 2002), rather it clustered with flagellated photosynthetic stramenopiles.

Morphologically, all species in this *Leukarachnion-Chlamydomyxa* clade are characterized by an amoeboid body form, plasmodia and net-like pseudopodia (i.e., reticulopodia) that form anastomosing networks, which is a characteristic of many Rhizarians (Cavalier-Smith et al., 2018). Yet, this clade is closely related to species of the Synurophyceae and Chrysophyceae, which comprise predominantly photosynthetic, mostly flagella-bearing stramenopile lineages and are not closely related to the Rhizaria (e.g., foraminifera, *Gromia*, *Corallomyxa*), which seem more closely related in a morphological sense. Together, these findings suggest that the formation of reticulate pseudopodia may not be a unique morphological feature for foraminifera and other related Rhizarian groups (e.g., radiolarians). Rather, it is likely that this group of *C. labyrinthuloides* + *Leukarachnion* sp. + *Synchroma grande* + Green Lake FFRP represents an evolutionarily independent lineage that is transitional between stramenopiles and Rhizarians. Resolving the evolutionary details of these relationships requires future dedicated phylogenomic study.

### 4.5. GL free-form reticulate protist: a candidate for microbialite fabric disruptor?

Because the FFRP were quite active, relatively numerous, and have far reaching and often extensive reticulopods, they could potentially remodel microbialite fabric on sub-millimeter scales.

The FFRP was noted in microbialites as well as off-platform sediments, commonly to depths of 3–4 mm in FLEC materials, a depth which is typically anoxic, or nearly so, in microbialites on a nightly basis, especially in summer. This shallow subsurface habitat may seem counterintuitive for a pigmented, presumably photosynthetic, organism. It is possible that the FFRP migrates to the microbialite surface to better expose its plastids to sunlight during the day given their observed rate of movement in Petri dishes. It is possible, also, for light to penetrate to >1 mm depth given reports that both carbonate and silicate minerals in stromatolites act as lenses (Kühl et al., 2003), and photosynthesis is supported at depth (Paterson et al., 2008). The presence of seemingly photosynthetic plastids in the FFRP suggests this taxon likely does not depend solely or even partly on heterotrophy for its nutrition. It is possible, however, that the FFRP endobionts are sequestered diatom chloroplasts, as known from a considerable number of marine foraminifera, some in habitats where sunlight exposure is ensured (Cedhagen, 1991; Jauffrais et al., 2018), while other kleptoplast-bearing foraminifers inhabit deep-water settings lacking sunlight (Bernhard and Bowser, 1999; Grzymski et al., 2002). Such occurrences of chloroplast-sequestering foraminifera in aphotic settings can coincide with anoxia exposure. These kleptoplastidic foraminifers can be anaerobic, using multiple metabolic pathways to allow inhabitation of such a seemingly “hostile” habitat (Gomaa et al., 2021; Powers et al., 2022). The role of such kleptoplasts remains unclear but the plastids seem to be integral to the survival of some foraminifera in aphotic anoxic sulfidic habitats (Gomaa et al., 2021).

*Chlamydomyxa labyrinthuloides* or an unknown species of *Chlamydomyxa* ASVs were detected on each of three sampling occasions from April to October, FFRP cells were microscopically observed on each of five sampling occasions, and FFRP or vacuolated cysts were noted in all three FLEC collections, suggesting they are present consistently on the microbialite platform, as well as episodically in the off-platform sediments. It may be important to note that we do not have any data besides geochemistry between November and April, so the presence and activities of the FFRP in winter remains unknown until future detailed studies are performed.

There seems to be some seasonality in the form of the FFRP, in that what we interpret as cysts (i.e., *C. labyrinthuloides* aplanospores) dominated the FLEC materials in September, while the amorphous (plasmodium) form dominated in November. Both cysts and the amorphous form occurred in May FLEC materials. While some of the morphological trends might be due to patchiness, it is also possible that the FFRP encysts in summer, perhaps due to warmer temperatures or due to the copious EPS covering. When the FFRP is encysted, the lack of reticulopodial activity may permit the EPS and/or microbialite layers to accrete appreciably due to the summer conditions when photosynthetic activity is highest.

### 4.6. Laminae disruption

In the cores without reticulopodial inhibition, the freshwater foraminifer *H. saranae* did cause detectable microbialite fabric disruption although this was not extensive. Because the foraminifer used in the disruption experiment was not native to GL, one may not be surprised that microbialite-fabric disturbance was minimal. There are multiple possible explanations for the modest disruption in our experiment. For example, the number of *H. saranae* introduced may have been insufficient or the experiment duration too short to cause substantial disturbance. Seeding of more *H. saranae* may not be realistic, as the general densities of the native and much smaller FFRP were not known at experiment initiation. Differences in the physicochemical parameters of GL lake water and the *H. saranae* culture water may have caused encystment or poor survival of the freshwater foraminifer. In particular, the slightly higher salinity (maximum value ~0.5–1 PSU, PTV unpubl.) and alkalinity of GL water may have been suboptimal for that species, limiting its activity. Similar experiments using the native *C. labyrinthuloides* are warranted.

### 4.7. Do the DMP stromatolites accrete?

The DMP microbialites differ from marine stromatolites because the former do not seem to accrete appreciably over time (i.e., the subsurface deeper than ~5–10 mm remains unstructured and massive). The DMP microbialites seem to have an annual cycle of layer development and obliteration. It is likely that the GL microbialite fabric remodeling occurs in late autumn or winter when we did not sample eukaryotes. To firmly establish if the DMP microbialites “recycle” materials rather than accrete, ^14^C-age dating should be employed, per other microbialite studies (Pace et al., 2018).

We suggest that, during winter, the metazoans will be sluggish due to low temperature, making them unlikely to be the lead cause of laminae disruption, and that the FFRP is the main cause of microfabric disruption. Evidence for this assertion is three-fold: (1) the relatively abundant FFRP have copious and far reaching reticulopods, like those known to rend biofilms (Bernhard and Bowser, 1992); (2) in the laboratory, they exhibit substantial (horizontal) migration rates, (3) heterotrophy can be induced in *C. labyrinthuloides* when light levels are low (Archer, 1875). Low light levels in GL are expected in winter due to the low angle of incident sunlight, short daylight periods, and, typically, ice cover. Once heterotrophic, the *C. labyrinthuloides* could rend the microbialites, thereby obliterating their layering or weakening the microbialite structure, causing laminae to become disrupted, as noted in our May samples. Such an assertion must be tested with dedicated analyses over appropriate time frames and physicochemical conditions. While the main cause for the decimation of stromatolite fossils in the Neoproterozoic remains debatable, our results do not discount a role for reticulopodia-bearing protists in the decline of freshwater stromatolites of any age.

## 5. Conclusion

The microbialites of the DMP platform of Green Lake near Fayetteville, New York support a considerable population of a reticulopodial-bearing protist, which is closely related to *C. labyrinthuloides*. We contend that GL *C. labyrinthuloides* reticulopodial activity during winter, when they are presumably heterotrophic, is responsible for lamina disruption on a moderate scale. Our contention should be tested with this native free-form protist under winter environmental conditions. Our observations are not mutually exclusive to other possible disruptors such as physical remodeling by metazoans or storm-induced wave activity.

## Data availability statement

The data presented in the study are deposited in GenBank, accession numbers OQ786765- OQ786773.

## Author contributions

JB, PV, and VR conceived of the study and secured funding. PV, LF, QM, and JB collected samples. LF, QM, LS, HY, FG, MR, PB-L, JB, PV, and VR analyzed samples. JB, VR, PV, LF, QM, LS, AL, FG, and AB contributed to data interpretations. JB drafted the manuscript with contributions from PV, VR, FG, AL, and AB. All authors contributed to the article and approved the submitted version.

## Footnotes

^1^ https://or.water.usgs.gov/alk/
