# Supplementary material for: Transition from stromatolite to thrombolite fabric: potential role for reticulopodial protists in lake microbialites of a Proterozoic ecosystem analog
Source: Front Microbiol. 2023 Oct 30;14:1210781. doi: 10.3389/fmicb.2023.1210781 (PMC10642914; doi:10.3389/fmicb.2023.1210781)
Supplement: Supplementary file 1 [file Data_Sheet_1.PDF]

Supplementary Material

Transition from stromatolite to thrombolite fabric: Role for reticulopodial protists in lake microbialites of a Proterozoic ecosystem analog

Joan M. Bernhard*, Luke A. Fisher, Quinne Murphy, Leena Sen, Heidi Yeh, Artemis Louyakis, Fatma Gomaa, Megan Reilly, Paola Batta-Lona, Ann Bucklin, Veronique Le Roux, Pieter T. Visscher

*** Correspondence:** jbernhard@whoi.edu

1 Supplementary Video

Supplementary Video 1. Time lapse clips of the GL Free Form Reticulate Protist with deployed reticulopodia moving in Petri dishes. Also visible are some of the native GL pennate diatoms. Scales and elapsed time noted in each of the three clips.

2 Supplementary Figures and Table

2.1 Supplementary Figures

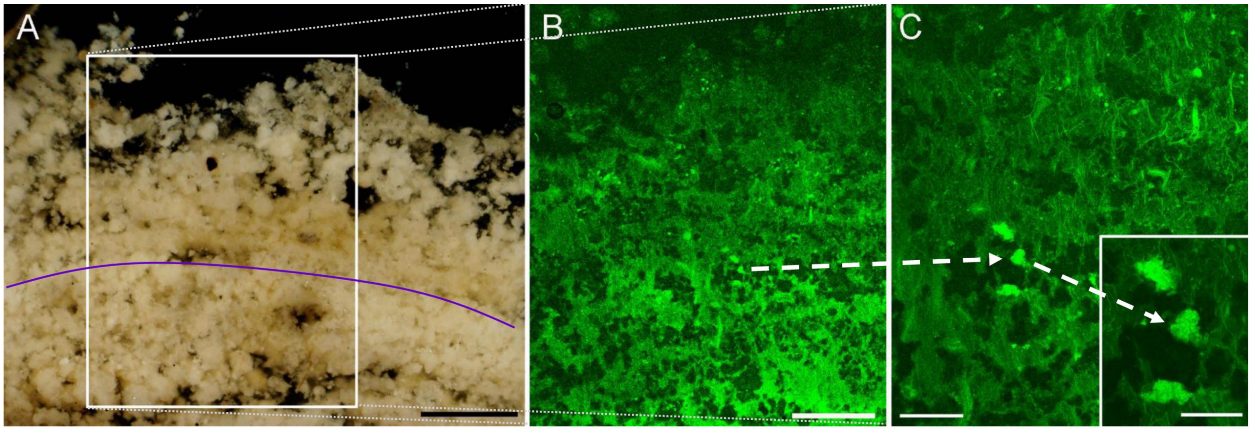

Supplementary Figure 1. Surface few mm of GL microbialite collected in May 2017, processed for FLEC. (A) Reflected light micrograph showing slight layering (immediately above purple line, which follows the arc) below overlying unconsolidated materials. (B-C) CLSM images (z stacks) of same FLEC section. (B) Highlighted portion of A (white box), revealing that slight layering is also evident in CLSM view. (C) Higher-magnification view of B showing the plethora of filamentous microbes, mostly aligned vertically and three amorphous cells (possible FFRP; dashed white lines link these cells through B,C and inset), which are shown in detail in inset. Number of images compiled/distance between images (µm) in B=5/2.0; C= 10/1.4; inset: 11/0.7 Scales: A = ~1mm; B= 600µm; C = 200µm; Inset = 100µm.

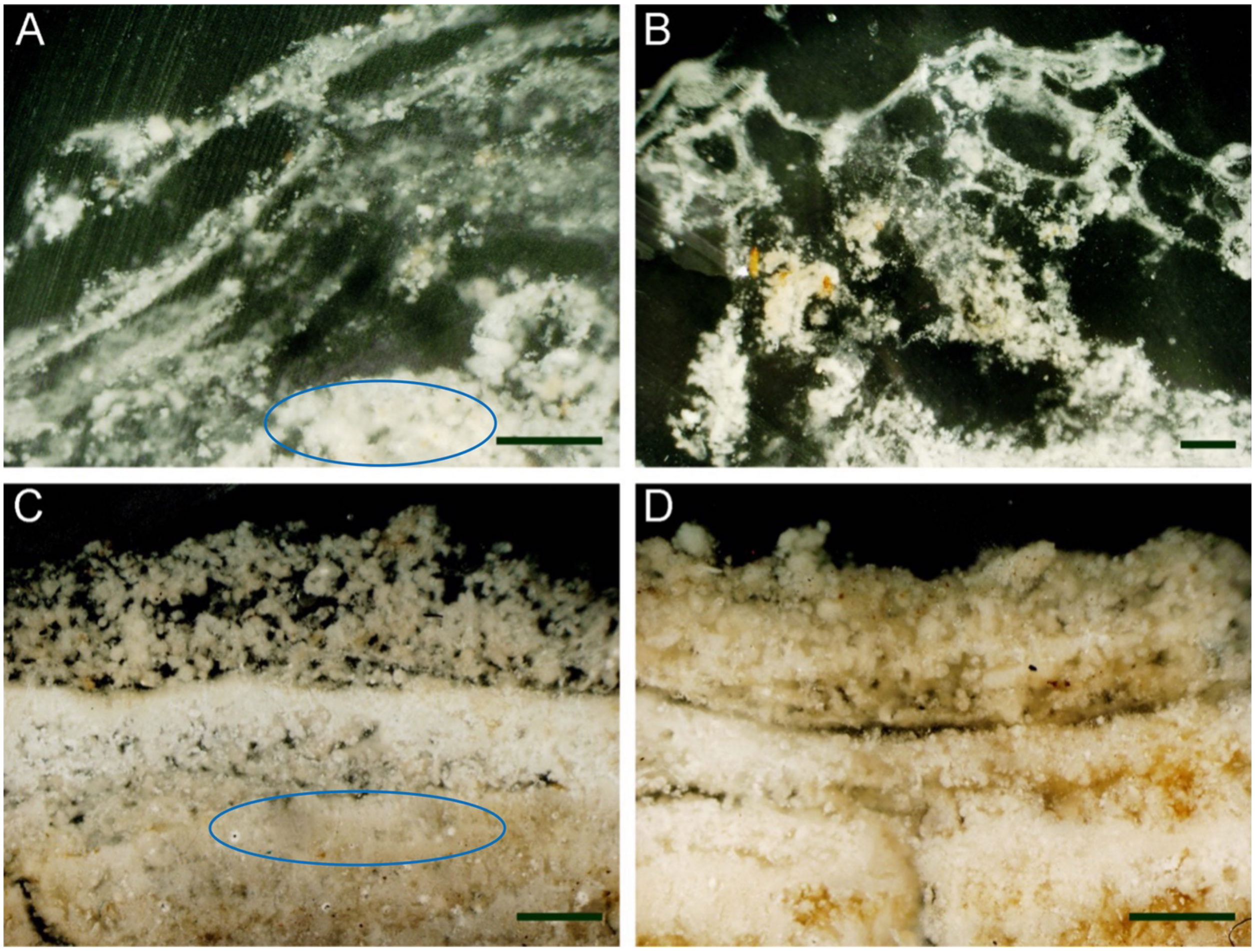

Supplementary Figure 2. Reflected-light micrographs of surface few mm of GL microbialites processed for FLEC, collected in September 2017 (A,B) and November (C,D). (A,B) Copious EPS overlying consolidated microbialite (ellipse in A). (C,D) Well-developed layers unerlying surficial unconsolidated materials. Crystalites, presumably of calcite (Brunskill, 1969) can be seen in ellipse of C. Scales: A-D = 1 mm.

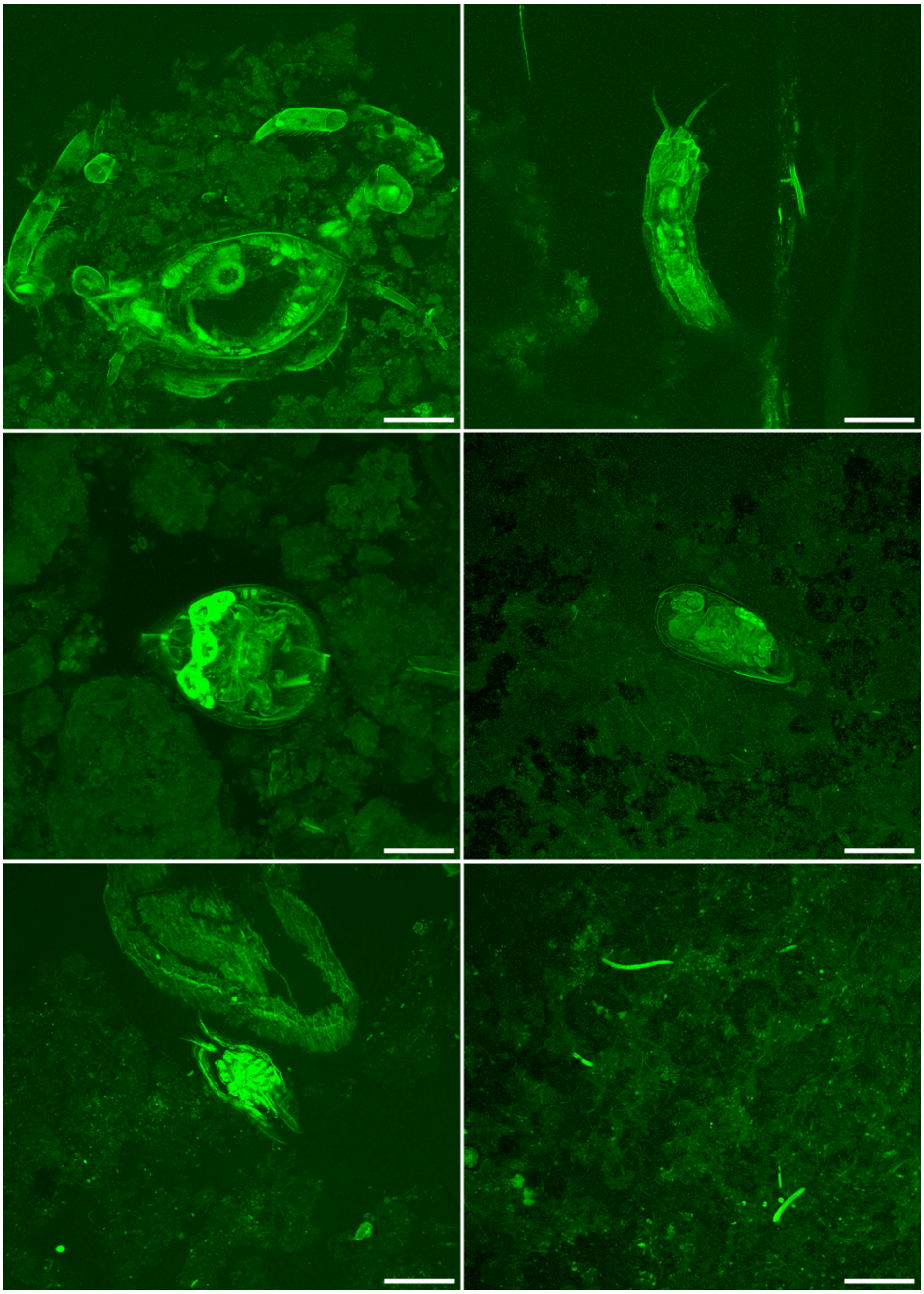

Supplementary Figure 3. CLSM image stacks of fluorescently labeled metazoans from GL sediments and microbialites. (A-E) Crustaceans. (A) Transverse section through a crab-like specimen from the surface mm of off-platform sediments. (B) Frontal section of tanaeid-like crustacean from top ~1-mm of off-platform sediments. (C) Ostracod in transverse section, from 3-mm depth in sediments. (D) Ostracod in sagittal section from 7-mm depth in microbialite. (E) Unidentified crustacean oblique section beneath multicellular algae, also in oblique section, from microbialite (~1mm depth). (F) Nematodes from ~5-mm depth in microbialite. Note presence of at least three nematodes among mostly fine filaments. Number of images compiled/distance between images (µm): A=71/1.4; B=66/1.4; C= 67/0.7; D= 33/1.4; E=27/1.4; F=39/1.4. Scales: A,B,D,E = 200µm; C,F = 100 µm. A-C collected in November 2019; B in September 2017; E,F in May 2017.

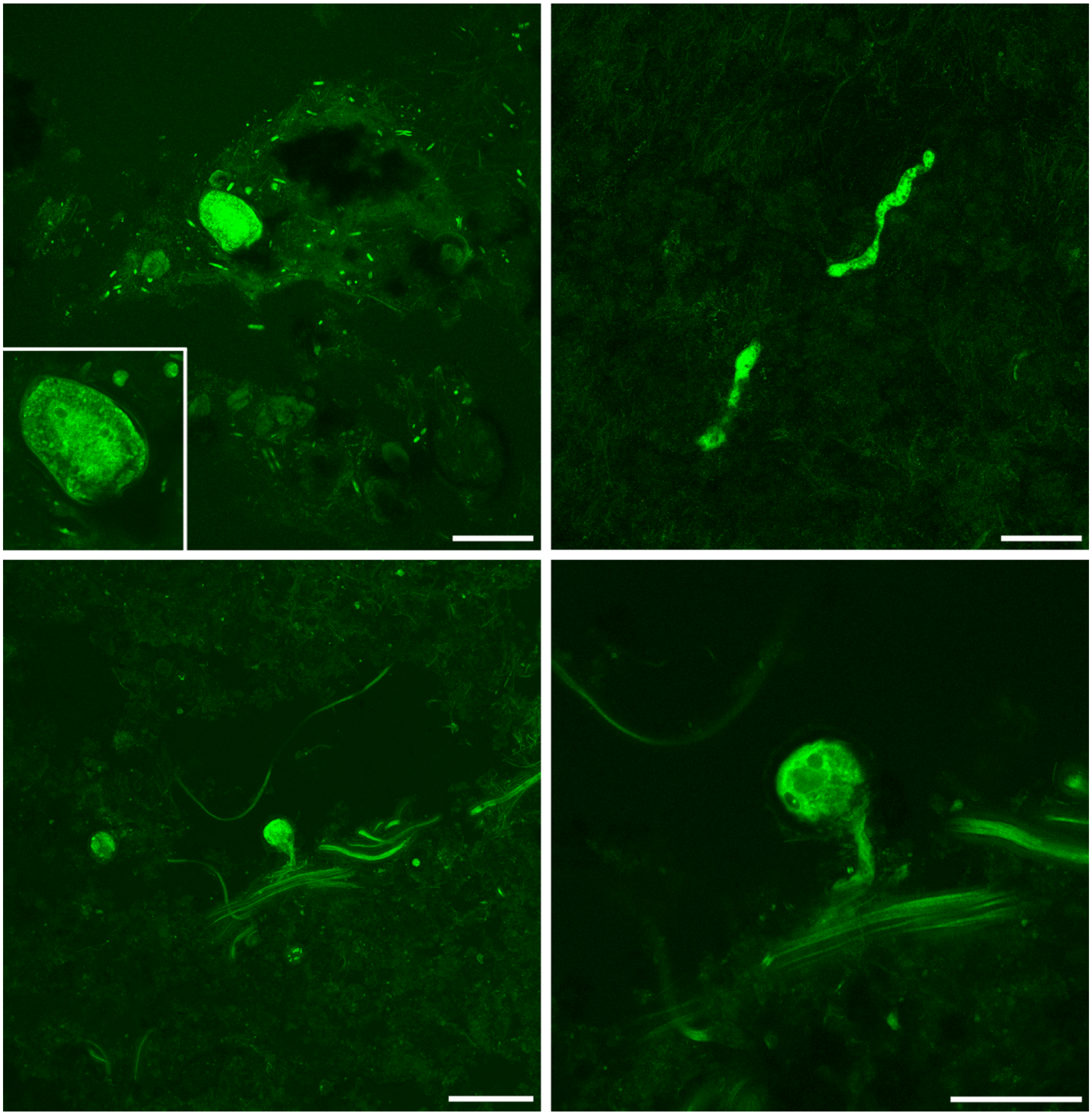

Supplementary Figure 4. CLSM image stacks of fluorescently labeled microbes from GL microbialites. (A) Ovoid vacuolated individual in surficial EPS, from Fig. 8C. Inset: Note non-fluorescent theca-like covering. (B) Well-vacuolated vermiform specimen from 2-mm depth. (C-D) Possible testate amoebae with vacuolated endoplasm and pseudopodia-like trunk extending toward image bottom, into filamentous entities. From 5-mm depth. Note non-fluorescent test around the main body in D. Number of images compiled/distance between images (µm): A=29/0.7; Inset in A = 1; B=23/0.7; C=26/1.4; D=1. Scales: A,B,D = 100 µm; C = 200 µm. A collected in September 2017; B in November 2019; C,D in May 2017.

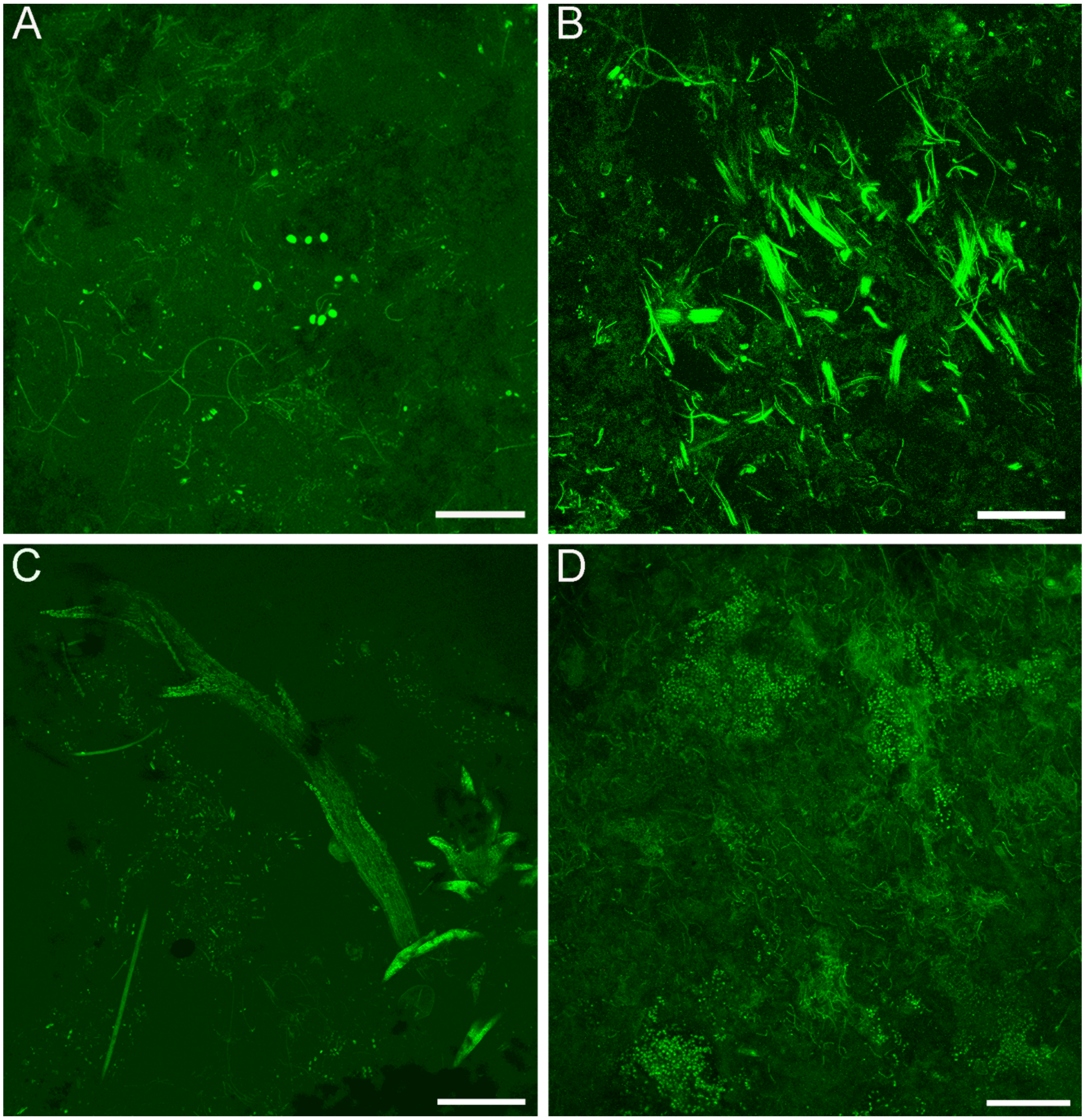

Supplementary Figure 5. CLSM image stacks of fluorescently labeled microbes inhabiting GL microbialites. (A) Small aggregation of possible flagellates, from 5-mm depth. (B) Area of filamentous strands, from ~ 12-mm depth, thicker than most observed in GL microbialites. (C) Macrophyte from top ~1-mm among EPS supporting many coccoid cells. (D) Co-occurrence of cocoid and filamentous microbes at ~ 4-mm depth. Number of images compiled/distance between images (μm): A=49/0.7; B=64/0.7; C=62/1.4; D=43/0.7. Scales: A,B,D = 100 μm; C = 200 μm. A-C collected in September; D in November.

2.2 Supplementary Table

Supplementary Table 1. Degree of anisotropy (DA) values for the six cores of the microfabric disruption experiment, presented by horizon category. "Top layer" refers to the sub-volume that was visually selected to represent the full microbialite layers (e.g., ~ 6 mm in Fig. 3C, green rectangle); "Uppermost layer" refers to the sub-volume that only encompasses the uppermost (e.g., ~ 1–2 mm, Fig. 3C lavender rectangle) of microbialite layers; "Whole sample" includes the full microbialite layers and the underlying indurated subsurface. To check for reproducibility of anisotropy results, the operation of visually selecting sub-volumes of top and uppermost layers was repeated at least three times in all samples. The small 2SD (two times the standard deviation of average) indicates that averaging 3 or 4 separate anisotropy calculations does not change the results. Core number label indicates treatment: those in italics had colchicine added; those in regular bold lacked colchicine. Potential DA values range from 0 to 1; the higher the value, the more anisotropic (layered) the measured volume was.

	**Core 0**		***Core 1***		***Core 2***		**Core 3**		**Core 4**		***Core 5***	
	after	before	after	before	after	before	after	before	after	before	after	before
uppermost layer 1	0.621	0.581	0.610	0.601	0.535	0.535	0.660	0.681	0.593	0.598	0.558	0.556
uppermost layer 2	0.604	0.589	0.611	0.607	0.543	0.537	0.657	0.672	0.585	0.590	0.547	0.554
uppermost layer 3	0.604	0.602	0.598	0.602	0.538	0.535	0.664	0.675	0.593	0.596	0.544	0.556
uppermost layer 4	0.597	0.570					0.658	0.678				
uppermost layer average	0.606	0.585	0.606	0.603	0.539	0.536	0.660	0.677	0.590	0.594	0.549	0.556
2SD	0.021	0.027	0.015	0.007	0.009	0.003	0.006	0.008	0.009	0.009	0.015	0.003
top layer 1	0.568	0.556	0.596	0.594	0.548	0.552	0.646	0.645	0.619	0.587	0.557	0.552
top layer 2	0.552	0.543	0.600	0.587	0.549	0.567	0.641	0.643	0.614	0.591	0.560	0.548
top layer 3	0.557	0.579	0.599	0.591	0.562	0.563	0.640	0.653	0.614	0.587	0.555	0.558
top layer 4	0.583	0.574			0.546	0.550						
top layer average	0.565	0.563	0.599	0.591	0.551	0.558	0.642	0.647	0.616	0.588	0.557	0.552
2SD	0.028	0.033	0.004	0.008	0.015	0.016	0.007	0.011	0.005	0.005	0.006	0.010
whole sample	0.507	0.507	0.519	0.516	0.531	0.531	0.527	0.529	0.540	0.552	0.534	0.533
